# GABA_B_ Receptor: Structure, Biological Functions, and Therapy for Diseases

**DOI:** 10.1002/mco2.70163

**Published:** 2025-04-16

**Authors:** Weijie Xie, Yuan Li, Xinyue Wang, Elena Blokhina, Evgeny Krupitsky, Marina Vetrova, Ji Hu, Ti‐Fei Yuan, Jue Chen, Hua Wang, Xiangfang Chen

**Affiliations:** ^1^ Clinical Research Center for Mental Disorders, Shanghai Pudong New Area Mental Health Center Tongji University School of Medicine Shanghai China; ^2^ Shanghai Key Laboratory of Psychotic Disorders, Brain Health Institute, National Center for Mental Disorders, Shanghai Mental Health Center Shanghai Jiaotong University School of Medicine Shanghai China; ^3^ Valdman Institute of Pharmacology Pavlov University St. Petersburg Russia; ^4^ Bekhterev National Medical Research Center for Psychiatry and Neurology St. Petersburg Russia; ^5^ ShanghaiTech University Shanghai China; ^6^ Co‐innovation Center of Neuroregeneration Nantong University Nantong Jiangsu China; ^7^ Department of Oncology The First Affiliated Hospital of Anhui Medical University Hefei China; ^8^ Department of Endocrinology Second Affiliated Hospital of Naval Medical University Shanghai China

**Keywords:** allosteric modulator, binge eating, GABA_B_ receptors, metabolism disorders, molecular signaling, psychiatric disorders

## Abstract

Gamma‐aminobutyric acid (GABA) B receptors (GABA_B_Rs) that acts slowly and maintains the inhibitory tone are versatile regulators in the complex nervous behaviors and their involvement in various neuropsychiatric disorders, such as anxiety, epilepsy, pain, drug addiction, and Alzheimer's disease. Additional study advances have implied the crucial roles of GABA_B_Rs in regulating feeding‐related behaviors, yet their therapeutic potential in addressing the neuropsychiatric disorders, binge eating, and feeding‐related disorders remains underutilized. This general review summarized the physiological structure and functions of GABA_B_R, explored the regulation in various psychiatric disorders, feeding behaviors, binge eating, and metabolism disorders, and fully discussed the potential of targeting GABA_B_Rs and its regulator‐binding sites for the treatment of different psychiatric disorders, binge eating and even obesity. While agonists that directly bind to GABA_B_R1 have some negative side effects, positive allosteric modulators (PAMs) that bind to GABA_B_R2 demonstrate excellent therapeutic efficacy and tolerability and have better safety and therapeutic indexes. Moreover, phosphorylation sites of downstream GABA_B_Rs regulators may be novel therapeutic targets for psychiatric disorders, binge eating, and obesity. Further studies, clinical trials in particular, will be essential for confirming the therapeutic value of PAMs and other agents targeting the GABA_B_R pathways in a clinical setting.

## Graphical abstract

Within the GABAergic system, GABA_B_Rs are potential therapeutic targets for dealing multiple metabolic and binge eating disorders and food addiction. Allosteric modulators and small peptides targeting GABA_B_R signaling pathways may be developed as attractive drug candidates that avoid the side effects of agents that directly bind these receptors.

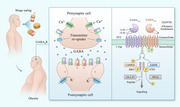

## 1 Introduction

Obesity is a growing problem worldwide, as over 2.1 billion people were overweight or obese in 2015 [1, 2]. China, in particular, has the highest number of affected individuals worldwide, with about 46% of adults and 15% of children being obese or overweight [3, 4]; the risk of these conditions is emerging as a major public health threat among children and adolescents, leading to considerable morbidity and mortality [1, 5, 6]. The central mechanism of weight gain or the development of obesity is an energy imbalance caused by complex interactions among an individual's genetic makeup [7], pathological stress [8, 9, 10], and environmental influences [6, 11, 12] or psychological factors [10, 13, 14, 15] in diet behaviors. At present, limited progress in the development of treatments for obesity have been made, and effective and widely accessible treatments for controlling weight gain in obese people need further development [6, 9, 16]. For instance, receptors of glucagon‐like peptide 1 (GLP‐1) and its agonists show great potential in treating obesity and overweight [17, 18, 19]. Given that obesity has a complex and multifactorial etiology involving pathological mechanisms and psychopathological mechanisms [13, 14], the existing approaches for sustained weight loss and weight gain prevention are relatively lacking.

Feeding behavioral patterns are primarily influenced by metabolic requirements and hedonic drive, especially when it comes to extremely calorie‐rich, sugary, or fatty foods [9, 20, 21, 22]. Overweight is mainly driven by overconsumption, binge eating (BE) of high‐calorie diets, and physical inactivity under various economic conditions [2, 6, 12, 16]. BE or overeating behaviors seem to be tightly associated with not only hedonic motivation [23, 24] but also natural addiction or craving for food [25, 26] and decision‐making or top‐down neural control [27, 28, 29]. Under normal conditions, hedonic or reward‐based eating, which involves food craving, seeking, and consumption, is well balanced and homeostatic [20, 29]. When an individual suffers from heavy social stress and a negative mood [9, 10, 30], severe pathological changes in the gastrointestinal environment [13, 14], metabolic abnormalities in periapical organs [31, 32, 33, 34], neuropathological disorders [35, 36], and dysfunction of brain circuit networks [20, 29, 37] other than those that regulate hunger and satiety [10, 29] can cause pathological ingestive behaviors [36, 38], BE [39, 40, 41, 42], compulsive eating [37, 43], and spontaneous eating [14, 26, 43], resulting in abnormal overeating or BE behaviors and food addiction. (Food addiction is a relative explanation concept that refers to the intense cravings, loss of control, and excessive consumption often associated with highly palatable foods [44].) These behavioral phenotypes notably resemble substance addiction‐like behaviors [25, 26, 45] and have long‐term negative consequences on physical and mental health [13, 14].

Obesity is partly caused by a food dependency that closely resembles a drug addiction, both in terms of behaviors and the neural processes involved [6, 13, 14, 46]. People who are obese may exhibit irregular eating patterns, such as increased indulgence in pleasurable eating, which partially contributes to excessive fat accumulation and a greater body mass [36, 38]. Based on the DSM‐5 criteria [47, 48, 49], bulimia nervosa (BN) and BE disorder (BED) patients show an amount of eating food without control in a discrete period, but BN is additionally characterized by recurrent inappropriate compensatory behaviors to prevent weight [48, 49]. BN and BED are prevalent mental eating disorders that impact approximately 4–5.8% of adults [50, 51]. These disorders are often accompanied by BE and food addiction behaviors out of control, which can contribute to obesity in 30–45% of individuals [13, 36, 52] and related metabolic disorders [7, 36, 38, 52].

Given the existing fact that the pathological influence and brain network dysfunctions of BE and food addiction‐related eating in the development of obesity, future treatments for overweight and obesity should specifically focus on the key molecules and neural circuits responsible for impulsive or compulsive overeating, and abnormal food craving [53], including psychotherapy and psychopharmacological interventions [54, 55, 56]. In the aspect of psychotherapy, cognitive behavioral therapy has been recommended as the vital treatment of adults with BN and BED, which can significantly improve the pathological features related to BE, also ameliorate the depressive and anxious emotion symptoms and boost their self‐esteem [54, 55, 57, 58]. Additionally, family‐based treatments, self‐help interventions, and digital interventions [54, 57] have some evidence of effectiveness and may be proposed to further develop in individuals with BE‐related disorders [54, 56, 57]. In the future, research on CNS‐targeting treatments should focus on the function of neurocircuits and molecular processes involved in BE and natural addiction to food [10, 14, 59, 60], such as reward sensitivity, impaired decision‐making, conditioning, and cognitive control, implying potential novel clues for interventions. Regulation of neurons and receptors involved in the GABAergic and glutamatergic systems could be novel pharmacological strategies for the treatment of food intake disorders [10, 42, 61], overeating [42, 62, 63], and obesity [64, 65]. Interestingly, the activation and regulation of GABA_B_ receptors (GABA_B_Rs) in both peripheral and central systems [64, 66] significantly contribute to the regulation of feeding behaviors and the development of food addiction (It is craving for palatable diets but could not be directly regarded as a critical definition of substance addiction [44].) and BE‐related behaviors [66, 67]. Evidence suggests that GABA_B_R could serve as promising targets for the treatment of BE‐like behaviors and food addiction [28, 64, 65, 68], contributing to prevent overweight and obesity.

Nevertheless, the safety and therapeutic efficacy of GABA_B_R‐targeting strategies on these mental disorders and feeding‐related disorders (including food addiction, BE, and obesity) in preclinical and clinical settings remain limited, insufficiently summarized, and only briefly discussed in the literature. Herein, our general review described the basic physiological structural features and functions of GABA_B_R in the CNS (Section 2); then summarized the different roles of GABA_B_R in the various neuro‐psychiatric disorders, including epilepsy, anxiety and depression, drug addiction, pain‐related disorders, schizophrenia, Alzheimer's disease (AD), and cognitive impairment (Section 3.1–3.7), and further explored the regulation effects of GABA_B_R‐targeting strategies on the feeding behaviors, BE or overeating, food addiction with impulsivity, overeating‐related overweight or obesity (Section 4.1–4.4), and others (Section 5). At last, this work fully discussed the treatment efficacy and the potential prospects of direct, partial, and negative activation of GABA_B_Rs and several regulatory sites associated with the GABA_B_R pathways (Section 6.1–6.4). In general, our comprehensive work contributes to identifying agents and clinical intervention strategies with good tolerability, great potential, and provide perspectives and insights for preventing and treating various neuro‐psychiatric disorders, food addiction, BE, and BE‐related metabolism disorders.

## 2 Physiological Structure and Functions

**FIGURE 1:**
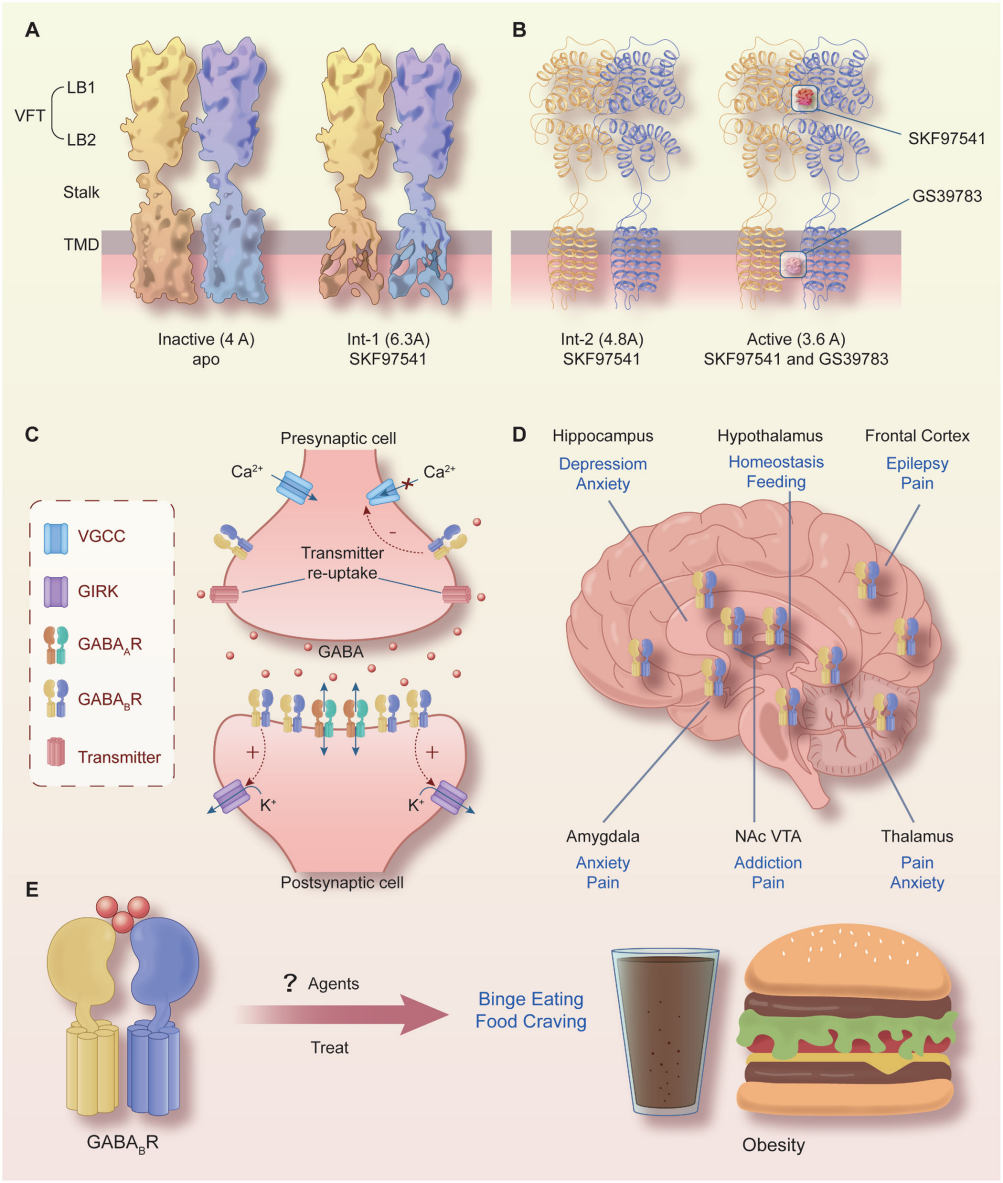
Structure, physiological functions, central distribution, and models of GABA_B_R heterodimers and their potential role in mental disorders. Maps (A) and models (B) of four different conformations of GABA_B_Rs, including active and inactive states and two intermediate conformations. The numbers in parentheses indicate the estimated resolution of the cryo‐EM maps. GB1 and GB2 are blue and yellow, respectively. The agonist (SKF97541)‐ and PAM (GS39783)‐bound state is the active state. (C) Possible sites of action of presynaptic and postsynaptic GABA_B_Rs. (D) The distribution of GABA_B_Rs in the brain and their underlying mechanism in neuropathologic diseases, including epilepsy, pain regulation, depression, anxiety and drug addiction, revealed by recent studies. (E) It remains unclear whether GABA_B_Rs represent a major pharmacological target for treating BE‐like behaviors, food addiction, and imbalanced diet‐related overweight and obesity.

GABA_B_Rs, which belong to the family of G‐protein‐coupled receptors, are metabotropic receptors that are widely distributed in the brain and body [69, 70]. X‐ray crystallography and single‐particle cryo‐electron microscopy studies [71] have proven (Figure 1A,B) that GABA_B_Rs consist of two subunits, GABA_B_R1 (GB1) and GABA_B_R2 (GB2), and function as obligate heterodimers [69, 71, 72]. With the development of artificial intelligence, the protein structure of GABA_B_Rs was predicted by Critical Assessment of Protein Structure Prediction and AlphaFold [73]. These valuable data offer crucial insights into the identification of biological processes linked to GABA_B_Rs, the structure‐based drug development, the design of interventions, and targeted mutagenesis.

**FIGURE 2:**
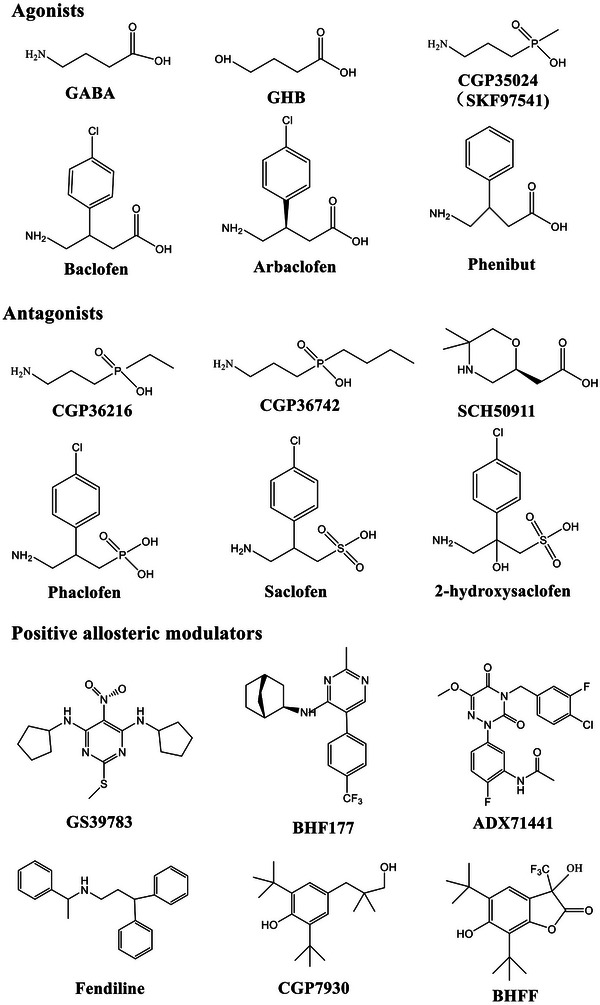
Chemical structures of representative full agonists, partial agonists, antagonists, and allosteric modulators of GABA_B_Rs. PAM is a positive allosteric modulator of GABA_B_R. The structures of these GABA_B_R‐targeting chemical compounds were verified by recent reports and study results.

As illustrated in Figure 1A,B, each subunit of GABA_B_R consists of seven helices that span the cellular membrane, and it contains an extracellular N‐terminus and an intracellular C‐terminus that are connected by both three extracellular loops and three intracellular loops [70, 72, 74]. GB1/GB2 is composed of an extracellular Venus flytrap (VFT) domain, a descending peptide linker, a seven‐helix transmembrane domain (TMD) and a cytoplasmic tail [69, 71]. When GABA or GB1 agonists/antagonists (Figure 2) bind to the VFT domain of GB1 (Figure 1A,B), a series of conformational alterations are initiated, leading to the transmission of transduction signals to the TMD of GB2, thereby initiating G protein signaling [72, 75]. On the other hand, the GB2 ectodomain does not bind GABA, but interacts with the GB1 ectodomain to increase agonist affinity by stabilizing the agonist‐bound conformation of GB1, and the binding of an agonist to the extracellular VFT of GB1 leads to G protein activation through rearrangement of the intracellular interface of the TMD of GB2 [71, 74, 75].

GABA_B_Rs, which are major inhibitory receptors, are present at both presynaptic and postsynaptic sites throughout the mammalian CNS [72, 74]. Presynaptically (Figure 1C), they inhibit neurotransmitter release by inhibiting Ca^2+^ channels; postsynaptically (Figure 1C), they generate low inhibitory postsynaptic potentials by activating inwardly rectifying GIRK channels [70, 71, 72]. Thus, GABA_B_Rs mediate slow and prolonged inhibitory neurotransmission and synaptic inhibition via indirect gating of neuronal K^+^ and Ca^2+^ channels and decreasing the levels of other second messenger targets (cAMPs) [70, 74]. Classical studies (Figure 1D) mapped the distribution of GABA_B_R binding site regions in the CNS, including the frontal cortex (medial prefrontal cortex, mPFC), the anterior olfactory nucleus [27, 76, 77, 78], the nucleus accumbens (NAc), the ventral tegmental area (VTA) [79, 80, 81], the interpeduncular nucleus, thalamic nuclei, different areas of the hypothalamus (mainly including lateral hypothalamus [LH], dorsomedial hypothalamus [DMH], ventromedial hypothalamus, periventricular nucleus, arcuate nucleus [ARC]) [63, 82, 83], the molecular layer of the cerebellum [76, 78], and the dorsal horn of the spinal cord [84]. Given the central roles of GABA_B_Rs in neurobiology and psychiatric disorders (Figure 1D) [70, 85, 86], it highlights the potential of the GABA_B_R system as a target for therapeutic intervention [85, 86, 87, 88, 89]. Therapeutic drugs that target GABA_B_Rs, such as baclofen (Figure 2), are used to treat various neurological diseases and pathophysiological feeding disorders, including epilepsy [75, 78, 90], pain [85, 86, 91, 92], depression [70, 77, 85, 93, 94], anxiety [85, 86], and drug addiction [89, 95, 96, 97]. For instance, baclofen has significant antiepileptogenic effects in spastic diplegia, multiple sclerosis [98], amyotrophic lateral sclerosis, and cerebral palsy [70, 72, 75, 85, 86] and exhibits great potential for treating alcohol use disorder (AUD) [88, 95, 96, 99], substance abuse (cocaine [100, 101], morphine [102, 103], and heroin [104, 105]), overeating or BE, and natural food addiction [66, 67, 70, 85].

Baclofen, as a clinically available representative agonist of GABA_B_Rs [89], has been clinically approved for the treatment of symptoms of spasticity, cerebral palsy, and multiple sclerosis [70, 72, 75]. SKF97541 (CGP35024 in Figures 1B and 2), a selective GABA_B_R agonist that was developed as the first analogue of baclofen, binds to the GB1 VFT domain (via orthosteric ligand recognition) but not to the GB2 VFT domain; following binding, agonist‐induced conformational signals in GB1 promote conformational changes in this subunit via its physical interaction with GB2, allowing functional responses by its cognate G protein [67, 71, 72, 74].

Furthermore, positive allosteric modulators (PAMs) bind the transmembrane region of the GB2 subunit (via allosteric ligand recognition) and strengthen the effect of agonists [59, 70, 71]. For instance, CGP7930 and ADX71441 (Figure 2), as PAMs and partial agonists of GB2, can facilitate agonist response at low concentrations and activate the receptor alone at higher concentrations [70, 72, 75] and have demonstrated remarkable preclinical effectiveness and tolerability in various models [70, 74, 85, 99]; by acting on presynaptic GABA_B_Rs and its GIRK/Kir3 channels, they inhibit cortico‐mesolimbic neurotransmission [70, 71, 74, 75, 85, 99]. PAMs with different structural folding patterns and increased potency, including GS39783, rac‐BHFF, and BHF177, have been developed [67, 71, 72, 74]; these agents exhibit more advantageous pharmacokinetics and efficacy compared with baclofen, including improved bioavailability, brain‐targeting ability, and reduced cytotoxicity.

Overall, the GABA_B_ receptor and its physiological roles are extremely complex, consequently, dysregulation of this receptor is involved in a broad range of neuro‐psychiatric disorders and feeding‐related disorders, and even obesity. As such the GABA_B_R are considered a highly attractive therapeutic target. The next work aims to discuss these issues and provide an overall assessment and comparative analysis of the potential of GABA_B_Rs as therapeutic targets in psychiatric disorders, nutrition and metabolism disorders, and feeding‐related disorders.

## 3 Effects in Neuro‐Psychiatric Disorders

### 3.1 Epilepsy

Epilepsy is the consequence of an imbalance between inhibitory and excitatory mechanisms within the brain. It is involved in alterations of classical neurotransmitters such as gamma‐aminobutyric acid (GABA), glutamate, serotonin, and neuropeptides in the hypothalamus and thalamus [106, 107]. Recent studies have exhibited that GABA_B_ receptors play a significant role in seizures in animal models and clinical trials [108, 109, 110, 111, 112, 113, 114]. GABAergic neurons pre‐ or postsynaptically inhibit epileptogenic neurons via GABA_B_ receptors, generating the low‐threshold calcium spike required to initiate burst firing, leading researchers to hypothesize [106, 107, 109]. In contrast, postsynaptic GABA_B_ receptors result in a more prolonged inhibitory postsynaptic current and are presynaptic, where they inhibit neurotransmitter release at inhibitory and excitatory synapses (Figure 1C and Table 1), thereby improving inhibitory and excitatory imbalances caused by epilepsy. Furthermore, some active compounds that target GABA_B_Rs enhance neural function and inhibit cell damage [115, 116, 117].

**TABLE 1:** Various compounds (agonists, antagonists, and allosteric modulators) targeting GABA_B_Rs and their related modulations in neuro‐psychiatric disorders.

Psychiatric disorders	Compounds	Effects	Mechanisms	References
Epilepsy	Antagonists: CGP55845 CGP62349 CGP 35348 SCH50911	**↓** Absence syndromes **↑** Learning and memory **↓** Hyperpolarization **↓** SWD occurrence **↓** Paired pulse depression	GABA_B_R: **↓** Neural hyperexcitability **↑** Hippocampal discharges **↑** Frequency of EPSCs **↑** Cell synaptic responses	[118, 119, 120, 121]
Epilepsy	Agonists: Baclofen GHB	**↑** Memory impairment **↓** Locomotor activity **↑** Hyperpolarization	GABA_B_R: **↓** Glutamatergic synapses **↑** Inhibition of transmission	[122, 123, 124, 125, 126]
Epilepsy	PAM: CGS 39783 BHF177	**↓** Aberrant hippocampal spikes **↓** Hyperexcitability	**↑** Presynaptic Ca^2+^ signaling **↑** Postsynaptic currents **↓** VCAM‐1, ICAM‐1, TNF‐α	[115, 116, 117, 124, 125].
Pain and analgesia	Agonists: Baclofen	**↓** Hyperexcitability **↓** Nociceptive responses **↑** Pain facilitation **↑** Analgesic effect. **↓** Membrane excitability **↑** Presynaptic inhibition	GABA_B_R: **↑** Rate‐dependent depression Spinal inhibition **↑** Kir current **↓** Cav2.2 channels **↑** GINIP, TREK‐2	[127, 128, 129, 130, 131]
Pain and analgesia	PAM: rac‐BHFF BHF177	**↓** Neuropathic pain **↑** Antinociceptive effects **↓** Trigeminal nociception	GABA_B_R: **↑** GABA_B_(1a) **↑** GABA_B_(2a)	[132, 133, 134, 135]
Anxiety and depression	Agonists: Baclofen CGP44532 SKF97541 2‐OH‐saclofen	**↑** Anxiogenic‐like effect **↑** Antidepressant effects **↓** Locomotor behavior **↓** Anxiety‐like effect	GABA_B_R **↓** c‐Fos **↑** GABA_B_R2 **↑** BDNF–TrkB **↑** Synaptic transmission	[136, 137, 138, 139, 140]
Anxiety and depression	PAM: CGP 7930 BHF177 GS39783	**↓** Anxiety‐like behaviors **↑** Anxiolytic activity **↑** Antidepressant activity	GABA_B_R **↑** GABA_B_R‐Kir channel **↑** Synaptic transmission	[133, 137, 141, 142, 143, 144]
Anxiety and depression	Antagonists: SCH 50911	**↑** Anxiolytic‐like effects **↑** Antidepressant activity	GABA_B_R: Cortical inhibition	[137, 145, 146, 147]
Drug abuse and addiction	Agonists: Baclofen SKF 97541 CGP 44532	**↓** Context‐induced reinstatement **↓** Self‐administration **↓** Cue‐induced reinstatement **↓** Active lever presses **↓** Morphine sensitization	GABA_B_R: **↓** DA neurons **↓** Dopamine release **↓** Reward enhancement Synaptic plasticity Neuronal excitability	[148, 149, 150, 151, 152, 153, 154]
Drug abuse and addiction	PAM: rac‐BHFF BHF177 ADX71441 GS39873 KK‐92A NVP998	↓ Self‐administration ↓ Ethanol drinking ↓ Reward effects ↓ Threshold lowering effect ↓ Negative aspects **↑** Antismoking	GABA_B_R: GABA_B_R1 D1 receptors Intracellular calcium Synaptic plasticity	[148, 155, 156, 157, 158]
Schizophrenia	Agonists: baclofen	**↑** Antipsychotic effect ↓ Behavioral hypersensitivity ↓ Cognitive impairment ↓ Negative emotion **↑** Prepulse inhibition	GABA_B_R: LICI DA release GABA release	[159, 160, 161, 162, 163, 164]
Schizophrenia	PAM: GS39783 CGP7930	**↑** Antipsychotic effect **↑** Social interaction **↑** Prepulse inhibition ↓ Cognitive impairment	GABA_B_R: Fos‐positive cells Neuronal excitability	[159, 160, 165]
Alzheimer's disease and cognitive impairment	Agonists: baclofen	↑ Spatial memory ↑ Learning ability ↓ Oxidative injury ↓ Neuronal apoptosis ↓ p‐Tau and Aβ formation	GABA_B_R: GABA_B_R–GIRK2 PI3K–AkT, MDA, SOD PKA–AAK1 pathway p‐Tau and Aβ formation Excitatory–inhibitory balance	[166, 167, 168, 169]
Alzheimer's disease and cognitive impairment	Antagonists: CGP 36742 SGS742 CGP55845 CGP52432	↓ Learning and memory impairment **↑** Memory function **↑** Synaptic functions	GABA_B_R: **↑** GABA_B_R/GIRK2 **↑** Glu, GABA and GABA_B_R **↑** ATF4/CREB2	[170, 171, 172, 173]
Autism disorder	Agonists: Baclofen STX209	↓ Autism‑like behaviors **↑** Novelty recognition ↓ Repetitive self‐grooming	GABA_B_R: **↑** GABA_B_R2 **↑** Spine density ↓ Network excitability	[174, 175, 176, 177]

Abbreviations: AAK1, adaptor‐associated kinase 1; CREB, CRE‐binding; EPSC, excitatory postsynaptic current; GHB, gamma‐hydroxybutyrate; ICAM‐1, intercellular cell adhesion molecule‐1; LICI, long‐interval intracortical inhibition; PAM, positive allosteric modulator; PKA, protein kinase A; SWDs, spike‐and‐wave discharges; TNF, tumor necrosis factor‐α; TREK‐2, two‐pore domain potassium channel; VCAM‐1, vascular cell adhesion molecule.

Previous evidence indicates that GABA_B_R activity can generate the low threshold calcium spike and initiate abnormal burst firings and spontaneous spike‐and‐wave discharges (SWDs), leading to these seizure‐related clinical symptoms [109, 122, 178]. In contrast, GABA_B_R antagonists suppress absence behaviors. For instance, GABA_B_R antagonists CGP55845A and CGP62349 markedly suppressed the development of absence syndromes to a greater degree, improved learning and memory retention and retrieval [118], and infusion of baclofen into the cerebrospinal fluid improved three distinct varieties of memory impairment [123]. The endogenous ligand gamma‐hydroxybutyrate (GHB) was reported to elicit a dose‐dependent reduction in locomotor activity [122]. However, CGP 35348 (GABA_B_R antagonist) reversibly antagonized GHB‐elicited hyperpolarization [119], and SCH50911 significantly reversed the changes in SWDs occurrence and locomotion induced by baclofen and GHB [122]. Moreover, CGP 62349 completely prevented hypothermia and the absence of seizures in both chemical models [120]. Further studies indicate that GABA_B_Rs regulate hippocampal neural hyperexcitability by inhibiting glutamatergic synapses [124, 125] and significantly reversing seizures and cognitive impairment.

GABA_B_R contributes to cortical and subcortical hyperexcitability and augments presynaptic calcium (Ca^2+^) signaling and postsynaptic currents in in vivo animal and in vitro cells or brain slices. First, CGS 39783 (a PAM of GABA_B_R) reduced aberrant hippocampal spikes in a mouse hippocampal kindling model [125]. The GABA_B_R antagonist CGP 55845 prolonged hippocampal discharges and increased spike incidences [125], attenuated the paired‐pulse depression of CA3 population spikes, and increased EPSC frequency in individual CA3 pyramidal neurons [124, 125]. Second, the presynaptic and postsynaptic functions of GABA_B_R were disrupted in hippocampal area CA1 in a chronic model of temporal lobe epilepsy [121]. In contrast, antagonists of GABA_B_Rs eliminated IPSPs and enhanced cell synaptic responses [121]. Third, GABA_B_R‐mediated cortical inhibition contributes to long‐interval intracortical inhibition [126]. GABA_B_Rs can potently inhibit transmission in tottering and reduce Purkinje cell output from the cerebellum of the Ca^2+^ channel‐mutant mouse [179]. In contrast, the abnormal alterations of GABA_B_R decreased paired‐pulse depression, caused neocortical hyperexcitability in epileptic WAG/Rij rat neocortex [178], and some variants impaired cell functions of GABA_B_R, perturbed neurotransmission by elevating presynaptic Ca^2+^ signaling [180]. Furthermore, glucose metabolism and AMP‐activated protein kinase can augment postsynaptic currents in thalamocortical neurons, regulate thalamocortical circuit excitability, and increase spike‐wave seizures, which are dependent on GABA_B_R cooperativity [181]. The positive modulation compounds of targeting GABA_B_R may reduce seizure‐related hyperexcitability.

Furthermore, some clinical and basic reports exhibit that GABA_B_R antibody‐related encephalitis may induce neuroimmune reactions and are accompanied by a stereotyped feature with epilepsy [112, 116, 117, 182]. A retrospective clinical study of GABA_B_R antibodies exhibited that the most frequent first symptom was recurrent seizures, amounting to 77% [111, 183]. In several antibody‐mediated encephalitis disorders, the epilepsy and cognitive decline risk is markedly high for disorders [111, 112, 116], including antibodies against NMDA, AMPA, and GABA_B_ receptors. Additionally, GABA_B_ receptor activation by BHF177 decreases VCAM‐1, ICAM‐1, and tumor necrosis factor‐α (TNF‐α) levels and relieves the inflammatory response in the hippocampal tissues of RE rats [115]. The underlying mechanisms are antibodies against neuronal surface antigens. The robust effects of antibody–antigen binding are involved in the inflammation‐related transcriptional and nontranscriptional pathways, resulting in hyperexcitability and contributing to synaptic function impairments [116, 117].

Epilepsy is a common neurological disorder characterized by recurrent convulsions and transient changes in consciousness. Targeting GABA_B_Rs is effective in treating partial‐onset and generalized seizures, can alleviate seizure‐related hyperactivity and hyperexcitability, and improves the deregulation of burst firing and neurotransmission (Table 1). Furthermore, potential antagonists or agents of GABA_B_Rs ameliorate synaptic dysfunction and inflammation, inhibit neuronal apoptosis, and protect against refractory epilepsy through the IRS‐1/PI3K/AKT axis [115].

### 3.2 Pain and Analgesia

GABA_B_Rs are vital for pain modulation and exhibit antinociceptive properties [113]. Regulating GABA_B_Rs affects intracellular signaling pathways through activation, thereby regulating neuronal excitability and inhibition [184]. Downregulating GABA_B_ receptors may contribute to the diminished inhibitory control of neurons in neuropathic pain models in rats and mice [113, 184, 185], diabetic neuropathy models, and acute inflammatory pain models [127, 132, 186, 187]. Current evidence (Table 1) has exhibited that GABA_B_R activation is involved in processing pain signals and analgesia induction in chronic pain conditions [185, 188, 189]. Specifically, the injection of GABA or GABA_B_R agonist through the brain ventricles can suppress neuropathic pain, demonstrating a dose‐dependent analgesic effect. This suggests that GABA_B_R activation can effectively reduce pain [113, 185].

Both activation of GABA_B_ receptor and baclofen produce inhibitory effects on hyperexcitability in response to natural stimuli [128, 129], reduce the mechanical allodynia in the neuropathic pain after spinal cord injury [129, 130, 131], and exert antinociceptive effects of subtherapeutic Pn3a in a model of acute postsurgical pain [131, 190]. Contrarily, PAMs of the GABA_B_ receptor can relieve neuropathic pain induced by chronic pain models with coapplication, including rac‐BHFF and BHF177 [133, 134]. Some compounds that regulate GABA_B_Rs also have antinociceptive activity in formalin and hot plate tests [132, 191], increase mRNA expression of GABA_B_(1a) and GABA_B_(2a) [188], enhance central descending inhibitory pain pathways, and suppress trigeminal nociception [135]. Moreover, GABA acting on GABA_B_Rs may decrease nociceptive responses [132], regulate somatosensory transduction [192], and enhance pain facilitation [127] during inflammatory sensitization and pain.

Further studies demonstrate that activation of GABA_B_Rs also regulates peripheral neuropathic pain, particularly in diabetic neuropathic pain. Painful diabetic peripheral neuropathy always involves damage to small neural fibers [187, 193], resulting in nociceptor hyperexcitability and dysregulation of synthesis and ionic channels [128, 187]. In contrast, the GABA_B_R agonist baclofen can mediate spinal inhibition, restore impaired rate‐dependent depression, and alleviate neuropathic pain in diabetic rats [194]. Intrathecal injection of baclofen significantly increased the paw withdrawal threshold in streptozotocin‐induced diabetic neuropathy rats [186]. It induced a significant reduction in spinal NR2B protein and mRNA levels (an NMDA receptor subunit) and downregulated the phosphorylated cAMP response element‐binding (CREB) protein levels [186, 195]. Additionally, GABA_B_R modulates the K^+^ (Kir) current in the trigeminal ganglia and increases the mean peak amplitude of Kir currents, suppressing trigeminal pain [128]. Baclofen can mediate gastric hypersensitivity and attenuate pain‐associated responses in a validated rat model of functional dyspepsia.

As for mechanisms, interactions of GABA_B_Rs with coupled Kir channels and some calcium channel proteins play vital roles in regulating neuronal excitability and neurotransmitter release. The activation of GABA_B_Rs can lead to the activation of G proteins, which in turn affect the opening of K^+^ channels (GIRK), thereby regulating the membrane potential and excitability of neurons (Figure 1C). For instance, the GABA_B_R agonist baclofen induces Kir currents [128, 196], GeXIVA (a 28 amino acid peptide) inhibits high‐voltage activated (HVA) N‐type calcium (Cav2.2) channels [197], reducing membrane excitability in mouse dorsal root ganglion (DRG) neurons, and reversibly potentiates inwardly rectifying Kir currents mediated by GIRK1/2 channels coexpressed with GABA_B_Rs in vitro [197]. On the one hand, baclofen significantly inhibits low‐voltage‐activated HVA Ca^2+^ channels and their currents in DRG neurons [198]. Contrarily, some peptides potently inhibit the Cav2.2 channels by activating pertussis toxin‐sensitive G_i/o_ proteins via the GABA_B_Rs [197, 199], such as analgesic α‐conotoxin Vc1.1 and GeXIVA, contributing to relieving peripheral pain. Moreover, GINIP, a G_ai_‐interacting protein, is involved in the mechanical hypersensitivity in the peripheral neuropathic pain [200, 201, 202]. GINIP deficiency can decrease baclofen‐evoked inhibition of HVA Ca^2+^ channels and impair presynaptic inhibition, thus blocking downstream signaling triggered by activation of G_ai_ proteins [203].

Furthermore, TREK‐2, a two‐pore‐domain potassium channel, can mediate leak Kir currents [204], and TREK‐2 can be enhanced following GABA_B_R activation, reducing neuron excitability in the trigeminal ganglion [205]. GABA_B_Rs inhibit the excitability of spinal dorsal horn neurons by regulating NMDAR, affecting synaptic input and function levels in diabetic neuropathic pain [186, 195]. Fibulin‐2 (extracellular matrix protein) also interacts with presynaptic GABA_B_Rs, regulates the presynaptic inhibition of neurotransmitter release, and weakens the GABA_B_‐mediated inhibitory effects [206]. An additional study has found that 14‐3‐3£ (a GABA_B_R1‐binding protein) interacts with GABA_B_R1 and impairs the downregulation of GABA_B_ signaling in the dorsal horn [207], and negatively modulate GABA_B_R signaling pathways by using related peptide inhibitors of Gαo_1_ and Gαi_1–3_ in Het mice [196].

These studies indicate that GABA_B_Rs can relieve or alleviate neuropathic pain via their mediated slow inhibitory neurotransmission and cross‐talk regulatory interactions with important GPCR channels or receptors, such as GIRK, NMDAR, Ca^2+^ channels, and TREK‐2 (Table 1). However, further research is needed on the mechanism of action and optimization of treatment regimens to reduce side effects [208, 209], such as motor function impairments.

### 3.3 Anxiety and Depression

Anxiety disorder is a common mental disorder characterized by excessive, persistent anxiety and worry that is difficult to control and significantly impacts an individual's daily life, work, and social interactions. Preclinical and clinical studies have demonstrated that alterations in GABA_B_Rs are involved in anxiety‐related disorders [210, 211, 212]. In animal anxiety models, the use of GABABR agents or agonists can significantly reduce anxiety‐like behaviors. Clinical studies have also explored the application of drugs related to the GABA_B_ receptor in the treatment of anxiety disorders and have already exhibited certain antianxiety effects via regulating the central GABAergic system, thereby reversing anxiety symptoms [113, 212, 213, 214]. Additional mechanisms are associated with presynaptic and postsynaptic inhibition regulation, excitatory transmission, neuroplasticity (Figure 1C and Table 1), and complex interactions with other neurotransmitter systems (such as 5‐HT) [215, 216, 217].

It is reported that CGP44532 (a GABA_B_R agonist) exhibits an anxiogenic‐like effect in several animal tests of anxiolytic activity [136]. The PAMs of GABA_B_R, including BHF177 [133, 141], CGP 7930 [137], and GS39783 [142], significantly increase time spent in the open areas and attenuate anxiety‐like behaviors [137, 143], displaying anxiolytic activity in behavioral experiments. Moreover, the differential modulation of targeting GABA_B_Rs may enhance the modulation of synaptic inhibition without significant effects on synaptic excitation and demonstrate a greatly reduced side effect [137, 143, 144]. Furthermore, SCH 50911 (a GABA_B_R antagonist) significantly produces anxiolytic‐like effects, while GABA_B_R agonists (baclofen and SKF97541) produce antidepressant and anxiogenic‐like effects [137].

Further studies suggest that GABA_B_Rs are also vital for modulating the anxiety‐related symptoms induced by ethanol and nicotine exposure or other mental disorders [138, 139, 140, 217, 218]. Baclofen (GABA_B_R agonists) markedly inhibited locomotor behavior in high‐anxiety rats that were exposed to single‐trial nicotine‐conditioned place preference [138] and abolished the rewarding properties induced by repeated nicotine administration [139]. Interestingly, the knockout of GABA_B_R1 subunit can block a global withdrawal score and an anxiety‐like behaviors caused by NIC withdrawal [140]. Besides, baclofen treatment alleviates the motor deficits and the elevated anxiety symptoms in BTBR mice [219] and rats with chronic cerebral hypoperfusion [220].

Regarding molecular mechanisms, it was found that the 2‐OH‐saclofen administration produced similar inhibition regulations of anxiety‐like effects and somatic manifestations and prevented the c‐Fos alterations induced by NIC [140, 217]. The activation of GABA_B_R2 could enhance the BDNF–TrkB signaling [220]. In GABAergic regulation, GABA neurons also depend on the modulation of synaptic transmission via the GABA_B_R‐Kir channel activation [144, 214, 221]. These studies demonstrate that GABA_B_Rs exert crucial roles in regulating anxiety disorders and may be developed as an important target for antianxiety treatment [211, 212].

In addition, some evidence indicates that the modulation of GABA_B_R is involved in the pathophysiology of depressive disorders. For instance, CGP 7930 and SCH 50911 exhibited antidepressant‐like activity in the forced swimming test [137], and baclofen can block the antidepressant‐like effect of ascorbic acid and ketamine [222]. Moreover, cortical excitability and inhibition functions were assessed through the established paradigms of paired‐pulse transcranial magnetic stimulation in patients with MDD [145, 223, 224, 225, 226]. The results demonstrated that different MDD subtypes may demonstrate different functions of deficient cortical excitability and inhibition related to GABA_A_Rs [224, 225], GABA_B_Rs [145, 146, 147], glutamatergic activity [147, 224], lower short‐interval cortical inhibition, lower cortical silent period, and higher intracortical facilitation [147, 226]. However, these existing reports are relatively limited, and further preclinical and clinical studies are needed to prove GABA_B_R modulation in MDD.

### 3.4 Drug Abuse and Addiction

Drug addiction is a chronic and relapsing brain disorder with significant social and health implications [227, 228]. The GABA_B_ receptor system has emerged as a crucial player in regulating drug addiction. GABA_B_Rs are widely distributed in the central nervous system and are involved in modulating neuronal excitability and neurotransmitter release [212, 229, 230]. In the context of drug addiction, drug abuse markedly disrupts the normal functions of the GABAergic system, leading to an imbalance between excitatory and inhibitory neurotransmission. Activation of GABA_B_Rs has been demonstrated to counteract some of the effects of drug addiction by reducing the reinforcing properties of drugs, attenuating drug‐seeking behavior, and modulating the neuroadaptations that occur in the brain following chronic drug exposure, ranging from alcohol [148, 149, 231, 232], cocaine [150, 233, 234], heroin [235, 236, 237] to nicotine [139, 155, 236]. The underlying mechanisms involve the regulation of various intracellular signaling pathways [228, 230, 238], modulation of synaptic plasticity, and interaction with other neurotransmitter systems such as the dopaminergic and glutamatergic systems [239, 240], ultimately influencing the neural circuits implicated in reward, motivation, and learning and memory processes that are dysregulated in drug addiction [229].

In the self‐administration patterns of alcohol drinking (AUD), re‐exposure to alcohol further induced the renewal of alcohol‐seeking behaviors in rats, increased the expression of Fos in the orbitofrontal cortex [151], the refusion of baclofen into the orbital frontal cortex (OFC) attenuated context‐induced reinstatement [151], and reduced alcohol self‐administration under the fixed ratio schedule of reinforcement [148]. Additional studies indicated that KK‐92A, rac‐BHFF, and ADX71441 are novel PAMs of GABA_B_Rs [156]; those treatments effectively reversed self‐administration [148, 155], attenuated ethanol drinking attenuated, and improved ethanol‐induced plasticity [155], which depicts nonsedative effects. Besides, SKF 97541 (a GABA_B_R agonist) dose‐dependently increased and decreased sensitivity to ethanol [149] in a Drosophila model exposed to alcohol, demonstrating that GABA_B_Rs also play a role in alcohol sensitivity and tolerance. Moreover, the gene promoter methylation of GABA_B_R1 demonstrated significant tissue‐independent changes with sex‐dependent differences in AUD. These studies indicate that GABA_B_R PAMs and agonists have high translational potential for treating patients with severe AUD.

In cocaine self‐administration and cue‐induced reinstatement, baclofen‐activating GABA_B_Rs dose‐dependently reduced the number of active lever presses in rats [150], weakened cue‐induced reinstatement [150, 152], and CGP 44532 (an agonist) significantly decreased cocaine‐induced reward enhancement in the brain stimulation reward paradigm, and inhibited the hedonic effects of cocaine [233]. Similarly, the novel GABA_B_‐positive modulator GS39873 and rac‐BHFF could modulate the behavioral effects of cocaine, attenuate the threshold‐lowering effect of cocaine administration, and thus suppress the rewarding effects of acute cocaine [157, 158]. Further clinical trials exhibit that baclofen may inhibit the earliest drug cue‐induced motivational processing [241]. Additionally, these mechanisms are engaged in inhibitory G‐protein‐dependent feedback pathways in the VTA DA neurons [242].

Besides, the baclofen administration significantly decreased the number of active lever‐pressing morphine self‐administrations, reduced morphine maintenance responses [153], and inhibited the development of morphine sensitization [154]. Moreover, treatment with baclofen decreased dopamine release in the NAc [154], altered nicotine‐rewarding properties in the conditioned place preference test [139], and reversed the negative aspects of nicotine withdrawal [217]. GS39783, BHF177, and NVP998 (allosteric modulators) also display the antismoking therapy nicotine self‐administration procedure and mediate GABA_B_R‐regulated signaling (intracellular calcium and ERK) [157]. Baclofen reduced heroin‐seeking behavior at doses [235] and ameliorated methamphetamine‐induced prepulse inhibition (PPI) deficits and object recognition memory impairment [243].

Overall (Table 1), agonists and allosteric modulators targeting GABA_B_Rs are vital for modulating drug addiction. By interacting with GABA_B_R, D1 receptors, and their related signaling in the central nervous system, they can influence neuronal excitability and neurotransmitter release. Specifically, GABA_B_R activation can reduce the reinforcing effects of addictive drugs by decreasing the release of dopamine in the reward pathways of the brain. This leads to diminished craving for the drug and the suppression of drug‐seeking behaviors. Additionally, they may modulate synaptic plasticity and correct neuroadaptations that occur with chronic drug exposure, potentially aiding in the prevention of relapse and offering a promising approach to treating drug addiction. PAMs seem to have a better pharmacological therapeutic index than GABA_B_R agonists; therefore, further exploration of the characterized, rewarding, and aversive stimulus effects of PAM application is required.

### 3.5 Schizophrenia

Schizophrenia is regarded as a severe mental illness characterized by cognitive impairment and olfactory dysfunction [244, 245]. The dysfunction of the GABA system has been linked to schizophrenia. Targeting GABA receptors, such as GABA_A_ and GABA_B_Rs, to enhance inhibitory neurotransmission is a potential approach [246]. Targeting GABA_B_Rs may help in reducing hyperactivity and improving symptoms related to abnormal neuronal excitability in preventing and treating schizophrenia (Table 1).

In MK‐801‐induced animal models, it could induce behavioral deficits in the positive, negative, and cognitive symptoms of schizophrenia; baclofen and GS39783 exerted a clear antipsychotic‐like effect on the behavioral deficits [159, 160], and SKF97541 (GABA_B_R antagonist) inhibited cAMP formation. Similarly, GS39783 (a PAM) effectively reversed MK‐801‐induced deficits in social interaction, forced swimming, and head twitch tests [247]. In prenatal PCP treatment‐induced adult mice, baclofen pretreatment significantly ameliorated behavioral hypersensitivity and PPI deficits [160, 165] and decreased the c‐Fos‐positive cells in the prefrontal cortex (PFC) [165] and PnC [160]. Moreover, baclofen dose‐dependently inhibited methamphetamine‐induced cognitive impairment [160, 161] and PPI impairment [160], and CGP7930 (a PAM) at doses that prevented ketamine‐induced deficits in PPI and decreased the potential in the hippocampus [248].

Clinical evidence demonstrates that GABA_B_Rs are involved in schizophrenia pathophysiology. GABA_B_R expression is reduced in pyramidal cells in Layer V in the entorhinal cortex and the inferior temporal cortex obtained from five patients with schizophrenia, as revealed by immunohistochemical assays [249]. In comparisons among 357 patients with treatment‐resistant schizophrenia and HC, the genetic variant analysis exhibited statistical differences for rs3749034 on GAD1 and rs10985765/rs3750344 on GABA_B_R2 [250]. The paired‐pulse TMS–EEG helps find that baclofen can induce a trend towards the enhancement of long‐interval intracortical inhibition, and GABA_B_R‐mediated cortical network abnormalities contribute to schizophrenia pathophysiology in patients [126, 162].

Moreover, GABA_B_R agonists can reduce this excitability via GABAergic inhibitory mechanisms, thereby improving gamma‐band responses [162]. Contrarily, NMDA‐stimulated GABA release and GABA_B_R activation modulate DA release in the brain by producing feedback regulation of dopamine transporter function via the related neural projections, such as the PFC‐NAc/VTA [164, 165]. Overall, GABA_B_Rs are part of the inhibitory neurotransmitter system and play a crucial role in schizophrenia prevention and treatment. Activation of GABA_B_ receptors can modulate neuronal excitability in key brain regions involved in schizophrenia pathophysiology, such as the PFC and the hippocampus. Enhancing inhibitory signals may help counteract the excessive excitatory activity often observed in this disorder.

Hence, GABA_B_R agonists could potentially improve the cognitive deficits associated with schizophrenia by regulating synaptic plasticity and neurotransmitter release. They might also impact reducing positive symptoms, perhaps by modulating the activity of neural circuits related to perception and thought processes. However, further research is needed to fully understand these mechanisms and develop more effective therapeutic strategies based on GABA_B_R modulation in schizophrenia.

### 3.6 Alzheimer's Disease and Cognitive Impairment

Alzheimer's disease is a progressive neurodegenerative disorder that primarily affects the brain and is characterized by a gradual decline in learning, memory, and cognitive functions [251, 252]. These cognitive impairments are accompanied by behavioral and psychological symptoms. Previous studies have specifically examined the role of GABA_B_Rs in cognition, learning, and memory processes under these conditions (Table 1). GABA_B_Rs are vital for synaptic plasticity, regulate neuronal excitability, and interact with other neurotransmitter systems to influence cognitive functions, hence being involved in the pathophysiological processes of Alzheimer's disease [113, 212].

In animal models, colchicine induction significantly impaired learning and memory ability in mice, whereas treatment with CGP 36742 (an antagonist) significantly inhibited learning and memory impairment [170, 171] and improved the levels of Glu, GABA, and GABA_B_R in the cortex and hippocampus [170]. Administration of SGS742 inhibits hippocampal CREB activity in rats by regulating the expression of transcription factors such as ATF4/CREB2 [171]. CGP55845 or CGP52432 (an antagonist) enhanced long‐term potentiation (LTP) in the Ts65Dn DG, improving synaptic plasticity [172].

In animal models of AD, baclofen can improve the spatial memory and learning ability of AD rats [166], activation of GABA_B_R reduces the oxidative stress injury (MDA, SOD, and GSH‐Px) [167], and suppresses the neuronal apoptosis, inhibiting p‐tau and Aβ formation by the PI3K/AKT signaling pathway [166]. Excessive Aβ induces the functional impairment of pyramidal neurons and their synaptic activation in hippocampus CA3, including the imbalanced excitatory potential and the decreased inhibitory potentials (IPSP). Concurrently, pharmacological modulation of the GABA_B_Rs can reverse the effects caused by Aβ [168], causing excitatory–inhibitory imbalance and neuronal death [166, 168, 253]. Moreover, partial modulation of GIRK2 channels can restore synaptic plasticity and improve impaired cognitive functions [168, 253]. Consequently, targeting GABA_B_R/GIRK2 signaling exerts a neuroprotective effect in AD models.

In other brain disorders accompanied by cognitive impairment, GABA_B_R function is altered. Under chronic cerebral hypoperfusion, baclofen markedly reversed the downregulation of GABA_B_R1, GABA_B_R2, and protein kinase A (PKA)–AAK1 pathways [169] and restored the balance of HCN1/HCN2 surface expression in rat hippocampal CA1, alleviating memory impairment and neuronal damage. In Down syndrome (DS), the functional parameters of GABAergic synapses are markedly disrupted, including the presynaptic release of GABA, IPSC, and postsynaptic GABAB/Kir3.2 signaling [173]. However, CGP55845 (GABA_B_R antagonist) ameliorates the deficient synaptic plasticity and learning. In epilepsy‐related cognitive impairment, GABA_B_R activation can reduce seizure‐induced cognitive damage, suppress excessive neuronal activity, and protect synaptic plasticity. For instance, CGP35348 (antagonist) improved the working memory and altered LTP.

The above evidence supports that GABA_B_Rs have great potential for treating cognitive impairment and Alzheimer's disease. GABA_B_R‐targeted drugs or modulators may be involved in modulating synaptic plasticity via the PI3K/AKT and CREB2 pathways, regulating neuronal excitability through G‐protein‐coupled signaling pathways (GIRK2, PKA, and CREB2), and exhibiting neuroprotective and anti‐inflammatory and antioxidant properties, promoting neuronal survival. These effects may slow the progression of cognitive impairment and AD by interacting with Aβ‐related processes. However, some reports are controversial in that either activation or inhibition of GABA_B_Rs improves cognitive function and prevents AD in various experimental models. The complexity is associated with both GABA_B_R and cognitive decline, implying that further detailed investigation is needed to confirm the precise roles of GABA_B_R in special AD and cognitive impairment models.

### 3.7 Other Mental Disorders

Besides the above summary, several reports exhibit that GABA_B_R plays an important therapeutic modulatory role in autism disorders. In the valproic acid‐induced autism model, treatment of STX209 (a GABA_B_R2 agonist) ameliorated autism‑like behaviors in the locomotion activity, sociability and preference, novelty recognition and marble‑burying via improving the spine density and GABA_B_R2 expression in the hippocampal DG /CA1 [174] and offspring of mice, prenatal baclofen administration significantly increased density of dendritic spines in the hippocampus and medial PFC, correcting the core autism‐like behaviors in F2 mice [175]. In BTBR and Fmr1‐KO mice models, R‐baclofen treatment can improve social scores [176] and reduce repetitive self‐grooming behaviors [176, 219]. Contrarily, baclofen illustrates no improvement effects on clinical autistic‐like features in patients with GABA_B_R1 and GABA_B_R2 gene variants [177]. Additionally, baclofen can restore GABA_B_R‐mediated inhibition and reduce network excitability in Tsc2^+/−^ mice [254]. It is found that neurexophilin‐1 can stabilize presynaptic GABA_B_Rs and postsynaptic GABA_A_R and improve synaptic short‐term plasticity, balancing transmission at excitatory and inhibitory synapses [177].

Some studies also have revealed significant pathological alterations in GABA_B_Rs and their mediated signaling pathways in Parkinson's disease [212, 255, 256], insomnia [256, 257], bipolar disorder [258, 259], and DS [172, 253]. Although dysfunction of GABA_B_R‐mediated inhibition may be involved in the development of these behavioral phenotypes, various strategies for targeting GABA_B_Rs, such as agonism, antagonism, and PAM, should be further investigated to confirm their therapeutic values and thus be developed as precise treatments for each neuropsychiatric and neurodegenerative disorder.

In general, GABA_B_Rs demonstrate excellent therapeutic promises in preventing and treating various mental disorders, mainly including epilepsy, anxiety and depression, drug addiction, pain‐related disorders, schizophrenia, Alzheimer's disease, and cognitive impairment (Table 1). On one hand, GABA_B_Rs can reduce the release of excitatory neurotransmitters through presynaptic inhibition, and regulate the hyperpolarization of the postsynaptic membrane via the GABA_B_R–GIRK channel currents, and thus contribute to maintaining the balance between excitation and inhibition. On the other hand, the special agonists and PAMs of GABA_B_R can interfere with the reward system by modulating the release of dopamine, influence the salience of stimuli and the formation of memories associated with rewarding or aversive experiences, and be involved in the psychiatric reward‐related learning, motivation, and cognitive processes in mental disorders, such as drug addiction. Additionally, the GABA_B_R‐triggered cAMP‐dependent signaling cascades are also involved in the regulation of the excitatory–inhibitory balance and synaptic plasticity, such as PI3K/AKT and its downstream signals (CREB2) in the PFC and other areas. More interestingly, these studies also reveal the special pathophysiological alternations of GABA_B_Rs and vital signaling pathways in different brain areas, and may exert different effects in each neuropsychiatric disorder.

There is another question that has been reported some unwanted side effects are produced by agonist or antagonists of directly targeting GABA_B_Rs, including tolerance, sedation, and motor impairment at higher doses. Hence, to avoid these undesirable effects, a unique class of neuroactivity agents should be developed to direct at special physiologic and pathologic processes involving GABA_B_Rs in the different neuropsychiatric disorders. Currently, allosteric modulators might represent a novel approach to have great potentials with fewer side effects. In the further, small allosteric modulators of targeting GABA_B_Rs will be further explored to highlight the therapeutic values in the treatment of mental disorders. In addition to the above‐mentioned brain psychiatric disorders, GABA_B_R‐associated small molecules also play a crucial role in regulating the pathological alternations and behavioral phenotypes involving both central and peripheral processes, such as abnormal eating and metabolism. In the next section, we will provide further detailed summary and discussions on uncovered roles of different GABA_B_R‐targeting strategies in nutrition and metabolism disorders.

## 4 Effects in Nutrition and Metabolism Disorders

Preclinical and clinical studies have indicated that the binding of agonists or antagonists to GB1/2‐regulating sites in GABA promotes or suppresses food intake or disordered eating [64, 66, 86] in the nutrition and metabolism disorders. However, the pharmacodynamics of GABA_B_R‐targeted agents in the treatment of BE‐related behaviors or disorders and obesity remain relatively lacking (Figure 1E), the treatment effects and therapeutic safety need to be further assessed and verified. It is imperative to further evaluate the regulatory effects of GABA_B_R‐targeting agents, including antagonists, agonists, and especially PAMs (Figure 2), on feeding, BE, food addiction, and obesity; analyze their effectiveness, safety, and therapeutic indices; and explore their mechanism, application prospects, and effects on signaling pathways downstream of GABA_B_Rs. Next, we mainly discussed the physiological regulations and pathological roles in the feeding and metabolism disorders.

### 4.1 Feeding Behaviors

**FIGURE 3:**
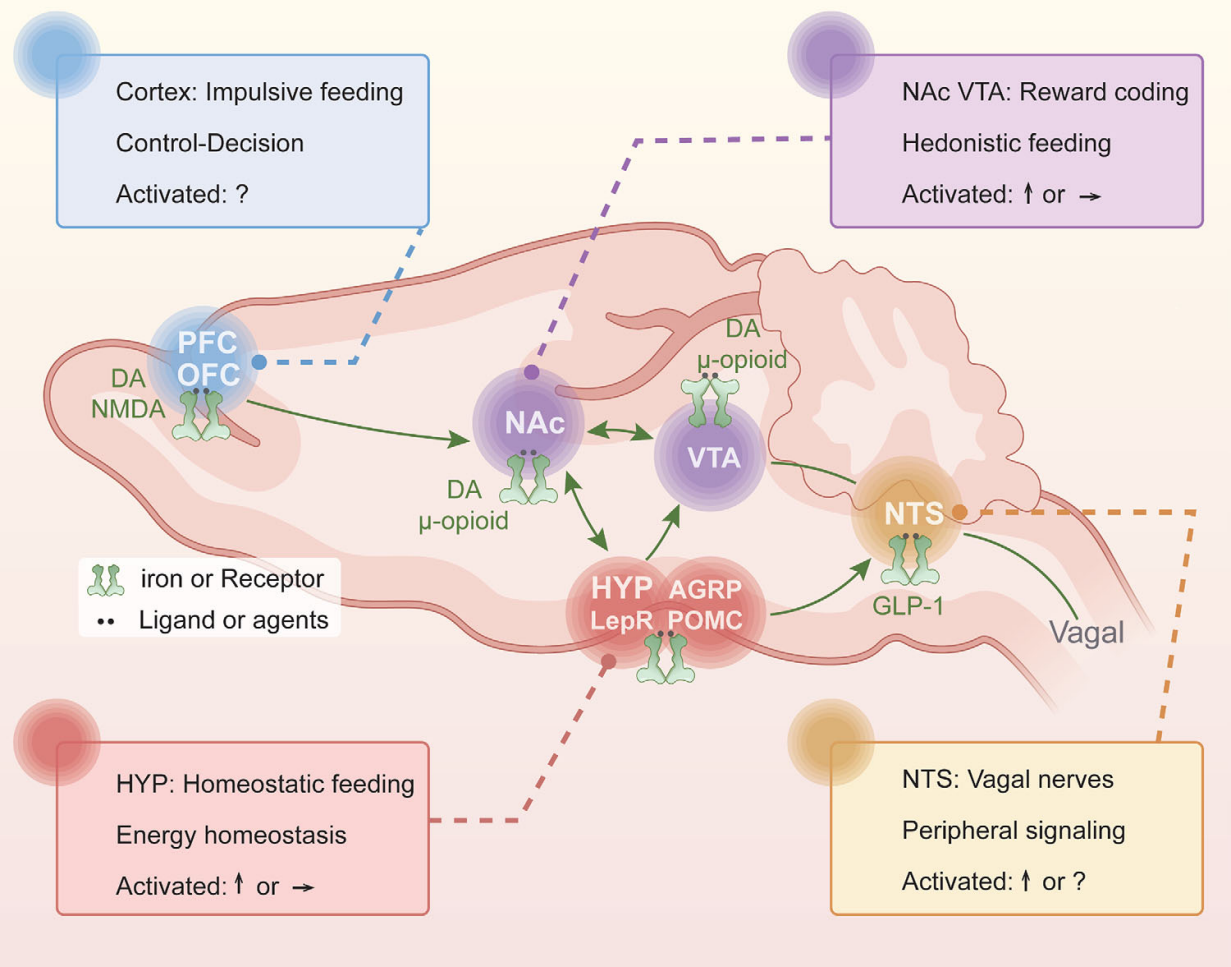
Neuromodulatory effects of GABA_B_Rs on normal feeding behaviors and related neural circuits in the brain different regions of CNS. Evidence has shown that activation of GABA_B_Rs in different regions of the CNS significantly regulates feeding behaviors under normal conditions.

The interplay of various brain networks is involved in homeostatic mechanisms versus nonhomeostatic in the ingestive behavior [13, 260]. The hypothalamus is as the primary brain satiety area within the homeostatic system that regulates food ingestion and energy balance [261, 262]; normal ingestive behavior is under the control of the extended reward network that mainly includes the NAc, VTA, and the substantia nigra, and is regulated by cognitive network regions, including the PFC [13]. Interestingly, GABA_B_Rs play a key role in feeding behaviors [67, 263, 264, 265], which also involves feeding neurocircuits and the different functional CNS regions that are mentioned above, mainly including the metabolic homeostasis‐related hypothalamic ARC, DMH and LH [61, 63], the reward‐related NAc and VTA [23, 29, 60, 86], and the PFC involved in decision‐making and executive control [23, 24, 29]. Based on the distribution of GABA_B_Rs in the different brain regions, we provided a comprehensive overview of the related neuromodulations and circuit pathways involving GABA_B_Rs in various brain regions and their impact on feeding behaviors; further discussed the specific effects and differences of GABA_B_R‐targeted agents on feeding and the underlying mechanisms (Figure 3 and Table 2).

**TABLE 2:** Summarized effects of targeted GABA_B_R or administration of baclofen on feeding behaviors.

Model	Compounds	Administration	Effects	Targeting	References
Male rats: normal diet	Baclofen	I.P.: 1, 4 mg/kg	↑ Food intake ‐ Normal diet	GABA_B_R	[266]
Rat: satiated normal diet	Baclofen	S.C.: 1–4 mg/kg	↑ Food intake	GABA_B_R	[267]
Pigs: normal diet	GABA Muscimol	ICV: 25–200 nmol	↑ Food intake	GABA_B_R	[268]
Male Wister rats: non‐deprived deprived‐food	Baclofen	I.P.: 1–4 mg/kg	↑ Nondeprived ‐ Deprived	GABA_B_R	[269]
Male SD rats: normal diet	Baclofen Muscimol	AcbSh: 0–876 pmol	↑ Feeding ↓ Food intake	GABA_B_R GABA_A_R	[270]
Male SD rats: imbalanced diet	NYP Muscimol Phaclofen	AIC: 2 nmol 79 pmol	↓ Thr‐basal diet ↓ Basal diet	Peptides GABA receptors ↓ Pyramidal cells	[271]
Lister rats: runway states normal diet	Baclofen	I.P.: 1–4 mg/kg	↑ Food intake:1 mg/kg ↑ Running speed ‐ Food intake: 2 /4mg/kg	Targeted GABA_B_R Complex appetites	[272]
Female rats: fasted, satiated	Muscimol Baclofen	Amygdala: CNA 0.05–1 nmol	↓ Palatable food ↓ Deprived of food	Activated GABA_A_R	[273]
Female mice: ovariectomized or normal diet	E2 DOI	ICV: 20 µM 50 nM	↓ Feeding	Desensitize ↓ GABA_B_R ↓ 5‐HT_1A_R	[274]
Male Wistar rats: nondeprived states peripherally	CCK Baclofen	IP: 5 pg/kg SC: 2 mg/kg	‐ Food intake ↓ Food intake ↓ Effects of CCK	↓ Release of CCK CCK: ↓ food intake	[275]
Male SD rats: satiated states normal diet	Muscimol Bicuculline LH: AP‐5	LH: 500 ng AcbSh:100 ng 10–200 ng	↑ Food intake ‐ Food intake ↓ AcbSh‐mediated feeding	NMDA Activated GABA_A_R Targeted GABA_B_R AcbSh–VPm–LH	[83]
Adult male Albino SD rats	Baclofen Saclofen Bicuculline	NAc shell/VTA: 200 ng, 1.5–5 µg 150 ng	↑ Feeding in VTA, NAc ↓ Baclofen‐elicited feeding	GABA_A_R, GABA_B_R With opioid receptors	[80, 276]
Male SD rats: normal diet	CGP‐35348 R‐baclofen CGP‐35348	LH:100–400 pmol 4.4–17.6 nmol 34–936 pmol	↑ Food intake ‐ Food intake	GABA_A_R Not GABA_B_R	[277]
Male SD rats: SA	Baclofen	LS: 1.7 nmol	↓ Anorectic effects	GABA_B_R	[278]
Male SD rats: normal diet	Bicuculline Muscimol Baclofen	VTA: 7.5–75 ng 200–500 ng 200 ng	Opioid: ↑ feeding in VTA Naltrexone: ↓ baclofen‐ induced intake	GABA_B_R Opioid receptors Coregulation in VTA	[279]
Male Wistar rats: vagally stimulate	Baclofen	I.P.: 2 mg/kg/day	‐ Food intake ↓ Effect of MC ↑ Gastric motility	GABA_B_R Vagal nerve stimulation	[280]
Male SD rats: nonfasted states	Baclofen	S.C: 1.0–4.0 mg/kg	↑ Food intake in free a paradigm for 90 min	GABA_B_R	[281]

Abbreviations: ↑, enhance or improve with significant differences; ↓, downregulate or inhibit with significant differences; ‐, no significant effects; AcbSh, NAc shell; AIC, anterior piriform cortex; CAN, central nucleus of the amygdala; CCK, cholecystokinin; ICV, intracerebroventricular; I.P., intraperitoneal; LS, lateral septum; MC, microchip stimulator; S.C., subcutaneous; SD, Sprague–Dawley.

#### 4.1.1 Metabolic Homeostasis: The Hypothalamus

The hypothalamus acts as a hub that integrates information from the CNS and peripheral signals with the external environment [13, 22, 60, 282], such as food availability and stress [10]. There is no doubt that GABA and GABAergic neurons in the hypothalamic feeding center play a role in promoting or regulating food intake [42, 61, 62, 63].

The existing evidence suggests that GABA_B_R neuromodulation in the LH improves feeding behaviors [10, 83, 277]. Microinjection of picrotoxin into the tuberal‐LH increased food intake, whereas microinjection of this drug into the anterior or posterior LH had no effect [277]. While neither blockade nor activation of GABA_B_Rs affected feeding behaviors in satiated rats, partial antagonism of GABA_A_Rs in the LH regulated feeding behavior [277]. Furthermore, injections of AP‐5 (an NMDA blocker) into the LH blocked the activation of LH neurons and suppressed NAc shell (AcbSh)‐mediated feeding [83]. In contrast, injections of bicuculline (a GABA_A_R blocker) or baclofen did not significantly affect food intake [83]. Injection of orexin A into the rostral LH induced feeding via GABAergic transmission, muscimol (a GABA_A_R agonist) significantly inhibited an increase in food intake, but baclofen showed no effects; in additions, in vivo GABA release within the rostral LH significantly coincided with orexin A‐induced feeding [283], indicating that projections from the LH to the NAc and GABAergic transmission may mediate feeding behaviors involving NMDA and GABA_A_R.

In addition, baclofen (a GABA_B_R agonist) significantly decreased NYP expression levels and significantly increased proopiomelanocortin (POMC) mRNA levels in the ARC in diabetic and diet‐induced obese mice [284]. Blood glucose and HbA1c levels decreased significantly and plasma leptin levels increased significantly increased in the baclofen groups, and the weight of epididymal WAT decreased significantly [284], indicating that GABA_B_R agonists decreased excessive adipose stores in obese subjects at least partially via the ARC. Direct injection of baclofen into the LH reduced the anorectic effects of stress and increased the consumption of sucrose [278, 285].

The LH and ARC are vital regions for integration of feeding information and have extensive connections with other hypothalamic and multiple extrahypothalamic brain regions [29, 65]. The current data indicate that orexin and GABAergic signaling within the LH and ARC is important for the regulation of appetite and feeding [286, 287]. It seems that orexin, GABA_A_Rs, and NMDA receptors but not GABA_B_Rs in the LH have a vital role in feeding regulation, but regulation of GABA_B_Rs in the ARC but not the LH is associated with overeating and the decreased anorectic effects induced by neuropathological states [285], demonstrating different effects in a brain‐subregion dependence manner. As GABAergic neurons in the LH coexpress GAD67, leptin receptor and melanin‐concentrating hormone [42, 61, 62, 63], GABA_B_Rs may interact with them, be mediated by projections to the LH [79, 288, 289, 290], and thus be compensated by these neuroprojections.

#### 4.1.2 Rewards Encoding: the NAc and VTA

The NAc and VTA, serving as major hubs of the brain reward circuitry, play crucial roles in the motivational and rewarding aspects of food seeking [291]; dysfunction of these reward processes may contribute to the pathogenesis of abnormal feeding and obesity [10, 292]. The GABAergic system and GABA_B_Rs may be involved in mediating feeding behaviors via a widely distributed reward network within the NAc and VTA [79, 292, 293, 294].

Microinjection of baclofen into the NAc shell or VTA significantly resulted in a substantial rise in food consumption above baseline levels [80]. In the NAc shell, GABA agonists decreased the firing rate of a population of local neurons and inhibited their neuronal activity [270]. Baclofen and muscimol (administration into the NAc shell, 0–876 pmol) significantly increased food intake and feeding at all doses [270]. In additions, an increase in local GABA levels elicited robust feeding in satiated rats [270]. These results demonstrate that activation of either GABA_A_Rs or GABA_B_Rs near the NAc shell and VTA is sufficient to increase food intake and that administration of endogenous GABA or baclofen into the NAc shell has a pronounced but specific effect on feeding behavior.

The interactions between GABA_B_Rs and GABA_A_Rs or opioid receptors occur within neural circuits between the NAc and VTA [80, 295, 296, 297, 298]. Pretreatment of saclofen (a antagonists, microinjection into the VTA) significantly decreased baclofen (microinjection into the NAc)‐induced food intake enhancement; following injection of saclofen into the VTA, injection of baclofen into the NAc did not significantly increase food intake, whereas following administration of saclofen into the NAc shell, injection of baclofen into the VTA affected food intake; moreover, preinjection of bicuculline into the VTA significantly decreased feeding induced by administration of baclofen into the NAc, whereas administration of baclofen into the VTA after preinjection of bicuculline into the VTA had no effects. Thus, although not to the same extent, induction of feeding by microinjection of baclofen into the VTA depended on the activity of GABA_B_Rs in the NAc shell [80], and induction of feeding by microinjection of baclofen into the NAc shell partially depended on the activity of GABA_B_Rs and GABA_A_Rs in the VTA [80].

In addition to GABA receptors, multiple selective opioid receptors in the VTA can significantly enhance feeding behaviors [276, 279]. DAMGO (an μ‐opioid agonist, microinjection into the VTA) increased food intake after 2 and 4 h, but this effect was altered by preinjection of an equimolar dose of bicuculline or saclofen into the same site in the VTA [279]. Injection of bicuculline alone into the VTA did not alter baseline food intake, whereas injection of muscimol alone into the VTA significantly increased food intake [279]. Furthermore, feeding induced by baclofen administration was significantly inhibited by preinjection of naltrexone into the VTA [279]; preinjection of a kappa opioid antagonist into the VTA significantly reduced the increase in food consumption elicited by injection of baclofen into the NAc shell [276]. Thus, GABA_B_ and μ‐opioid receptors in the VTA and NAc coregulate feeding [279], indicating the presence and coregulation of GABAergic and enkephalinergic neurons in both regions.

The existing results above imply that GABA_B_Rs and GABA_A_Rs are involved in the regulations of feeding behaviors, and obviously interactions with intact opioid receptors, mainly mu and delta opioid receptors [276]. Activated opioid receptors inhibit local GABAergic interneurons in the VTA and NAc, and opioids act through mu and kappa opioid receptors in the VTA. GABA_B_R and opioid receptor signaling in the VTA and NAc can synergize and engage in crosstalk to mediate feeding behaviors (Figure 3 and Table 2).

#### 4.1.3 Control and Decision: the Cortex

Motivating factors, emotional cues, and certain cognitive functions can play important roles in overeating or BE, food addiction, and obesity, which are related to the functions of the ventral striatum, PFC, and OFC [64, 77]. Control and decision‐making‐abilities related to the urge to eat desirable food vary among individuals and might be one of the factors that contribute to vulnerability to overeating, food addiction, and obesity. Currently, there is limited evidence for whether GABAergic neurons and GABA receptors in the cortex play crucial roles in feeding behaviors [27, 77, 78]. Herein, we further discussed the regulation of targeting GABA_B_R in cortex on food consumption.

Under food‐restriction conditions, neural processing and neuromodulation in the anterior piriform cortex (AIC) and ventromedial PFC (vmPFC) regulated palatability‐driven feeding, motivation, and the duration of individual feeding bouts [27]. Injecting muscimol into the AIC significantly reduced the consumption of food, the overall duration of feeding, and the average duration of feeding on palatable food. In contrast, injection of muscimol into the vmPFC markedly increased the feeding bout duration, did not impact the total consumption of food (either chow or a chocolate shake), and significantly reduced food exploration‐like behavior [27]. Microinjection of bicuculline into AIC elicited the intake of an amino acid‐imbalanced diet, and administration of phaclofen into the AIC decreased the intake of basal diets but did not affect the consumption of an imbalanced diet, implying that GABAergic receptors in the mPFC and AIC in the cortex may mediate abnormal intake of an imbalanced diet [271] and HFD [64], at least in part. Furthermore, GABA_A_Rs and possibly GABA_B_Rs affected the intake of an amino acid‐imbalanced diet [273]. Direct injection of baclofen into the lateral septum (LS) decreased the anorectic effects of stress and increased sucrose intake [278].

GABA_B_Rs in the AIC and mPFC of the cortex have a crucial role in the selection of food and intake of an amino acid imbalanced diets or HFD [64, 271]. mPFC and OFC neurons are involved in decision‐making and behavior driven by reward and motivations [13, 299, 300]. If the cortex is affected by an imbalance between encoding reward value and control‐making decisions, “top‐down” (cortical) regulator functions become imbalanced, similar to substance use disorders (SUDs) [301]. Therefore, GABA_B_Rs in the cortex may have a significant role in the treatment of abnormal feeding, BE, and food addiction via blockade of “top‐down” inhibition, which need to be fully explored (Figure 3).

In general, administration of a GABA_B_R agonist or activation of GABA_B_Rs can induce feeding of a normal diet in a normal state (Figure 3 and Table 2), but it has also shown that GABA_B_Rs have complex effects on feeding behaviors through different motivational mechanisms, under different environmental conditions, and via reactions of different intensities (Figure 3). In additions, GABA_B_Rs may interact with other neuropeptides (CCK), neurotransmitters (DA) or other related receptors to regulate food intake through different mechanisms as shown in Table 2 and Figure 3. These roles of GABA_B_Rs demonstrate some differential dependence on its expression in different brain regions, including the metabolic homeostasis‐related hypothalamus, the reward‐related NAc and VTA, and the decision‐making‐related cortex, and its involvement in the different pathological conditions and interactions with other signals, which would be further discussed in the following sections.

### 4.2 Binge Eating

**FIGURE 4:**
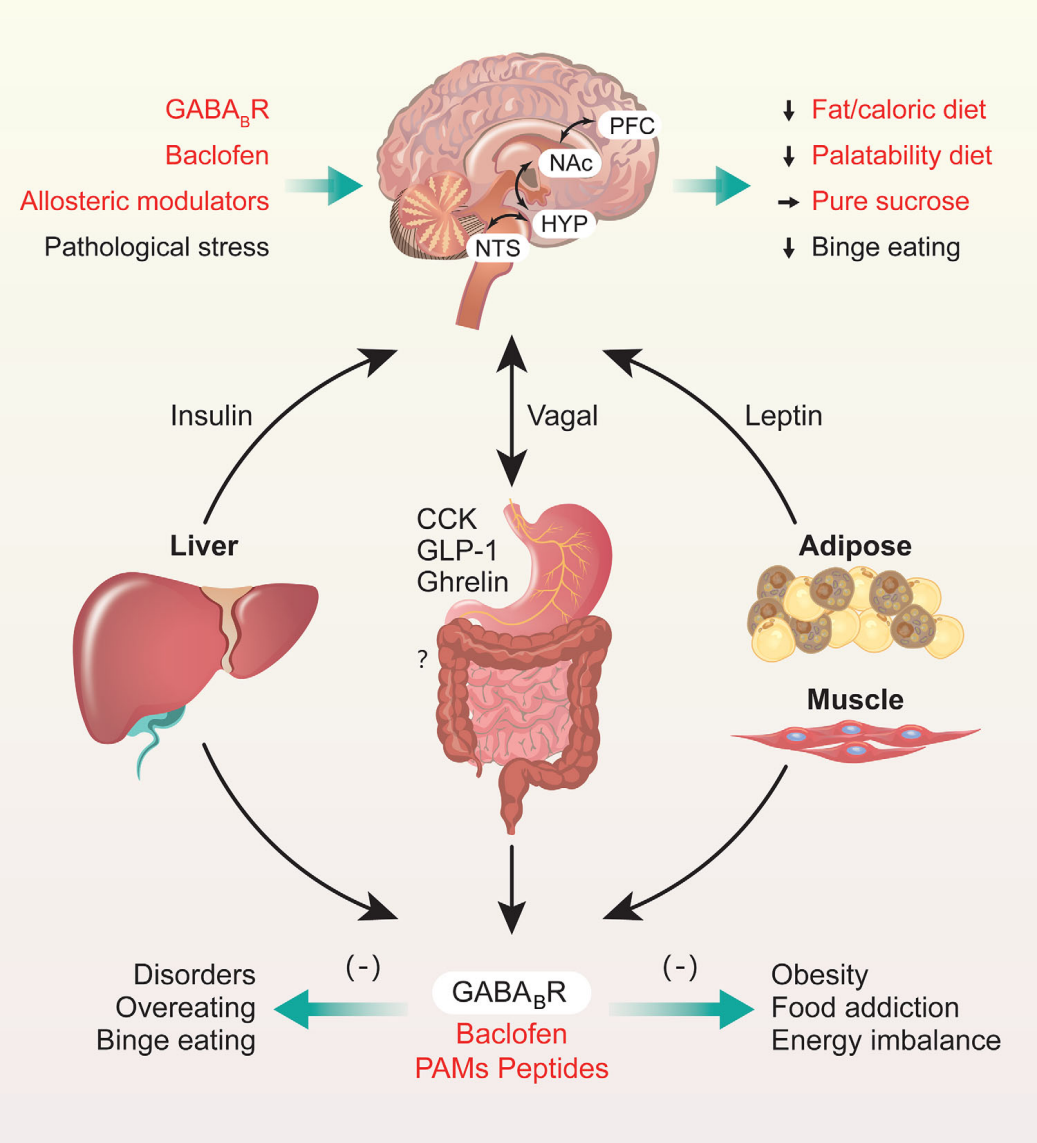
Effects of GABA_B_Rs on binge eating, food addiction, and obesity through central and peripheral regulation. On one hand, GABAergic neurons and GABA_B_R may play a vital role in the regulation of energy homeostasis and feeding via the central hypothalamic (leptin, MCH, and CCK) and reward systems (NAc and VTA) and inhibition of food craving, reward abnormalities, and control‐decision imbalance in feeding behaviors (mPFC/OFC); on the other hand, regulation of GABA_B_Rs may be associated with peripheral vagal neuromodulation and regulation, which needs to further explore. These findings imply the potential of GABA_B_R‐targeting strategies in the treatment of BE, food addiction, and obesity.

These results above suggest that GABA_B_R activation can increase intake of a normal diet in a normal state (Figure 3 and Table 2) but have also shown that GABA_B_Rs have complex effects on appetitive behaviors that may interfere with its effects on feeding behaviors, as shown in Table 3 and Figure 4. Strategies targeting GABA_B_Rs may regulate overeating/BE feeding behaviors and eating‐related disorders [66, 67], but this effect strongly depends on the pathological alternations and models and remains to be verified. Here, our next step mainly discussed the effects of GABA_B_Rs for overeating and BE.

**TABLE 3:** Summarized effects of GABA_B_Rs on binge eating behaviors, food addiction for palatable food, and its related obesity through central nervous and peripheral regulation.

Model	Compound	Administration	Effects	Targeting	References
Male SD rats: Fat emulsion Palatable diet Sugar–fat emulsion	Baclofen Naltrexone	I.P.: 1, 1.8 mg/kg 0.1, 1.0 mg/kg	↓ Palatable food intake ‐ Palatable food or chow Combination: ↓ Palatable diet	GABA_B_R	[26, 302]
Male SD rats: Non‐food‐deprived Limited access	Baclofen SCH 23390 Raclopride	I.P.: 0.6–1.8 mg/kg 0.03–0.3 mg/kg 0.03–0.3 mg/kg	↓ Intake of shortening of binge fat or sucrose ‐ Sucrose, chow intake	GABA_B_R opioid receptors D1, D2 receptors	[303, 304]
Female Wistar rats: Intermittent access Chocolate food	LDX SB‐334867 R‐baclofen	PO: 0.1–1.5 mg/kg I.P.: 3–30 mg/kg I.P.: 1–10 mg/kg	↓ Chocolate intake ↓ Chocolate intake ‐ Chow, water intake	Orexin‐1 GABA_B_R D1 receptor α1‐adrenergic	[305]
Male SD rats: Fat/sucrose mixtures Limited access	Baclofen; Naltrexone Raclopride	I.P.: 0.6–1.8 mg/kg 0.03–0.3 mg/kg 0.03–0.3 mg/kg	↓ FSM intake ‐ 32% FSM Sucrose concentration	GABA_B_R D2 receptor mu‐receptor	[304]
Male SD rats: Limited access Solid fat emulsion	Baclofen Raclopride	I.P.: 0.6–1.8 mg/kg 0.03–0.3 mg/kg	↓ Emulsion intake ↓ 32%, 56%‐fat emulsion ↓ 18%‐fat emulsion; ↓ Fat emulsion in daily	GABA_B_R D2 receptor Fat concentration mu‐opioid receptor	[263]
Male db/db mice: High fat diet‐HFD	Baclofen SKF 97541 3‐APPA	OA: 4, 100 × 10^−6^ M 10 × 10^−6^ M 10 × 10^−6^ M	↓ Food intake ↑ Plasma leptin ↓ Epididymal WAT ↓ Blood glucose, HbA1c	NPY GABA_B_R Energy balance Adipose stores	[306]
Male SD rats: Fat chow Sweet–fat chow	Baclofen	I.P.: 0.6–1.8 mg/kg	↓ Vegetable‐fat binge ↑ Sweet–fat binge ‐ Sucrose, standard chow	GABA_B_R Galanin Pure fat intake	[307]
Male SD rats: SA, solid‐fat Non‐food deprived	(R)‐baclofen	I.P.: 0.3–1.8 mg/kg	↓ Pellet in SA ↓ Shortening responding	GABA_B_R	[308]
Male SD rats: Limited access Lard intake, binge‐type	(R‐S)‐baclofen	I.P.: 0.0–1.8–3.2 mg/kg	↓ Lard intake ‐ Chow intake	GABA_B_R Food type conditions	[68]
Male SD rats: Under binge‐type	(R)‐baclofen	I.P.: 0.3–1.8 mg/kg	↓ 2‐h shortening intake no effect	GABA_B_R activation	[309]
Obesity people: Women, men	(R)‐baclofen	OA: 25 mg on day 7 30 mg on day 10	↓ Body weight, waist ↓ Serum leptin ↓ Appetite and desire for sweets	Activation of GABA_B_R Adipose Regulation	[310]
Binge eating: Women	Baclofen	OA: 60 mg/day for 10 weeks	↓ Binge frequency ↓ Cores of the Food Craving Inventory‐II	Targeting GABA_B_R	[311, 312]
Male SD rats: Restricted feeding Palatable WD diet	Muscimol: VMN‐106.8 ng NAc‐5.7 ng	Baclofen: VMN‐0.25 µL/side NAc‐0.5 µL/side	↓ WD intake during the subsequent 15 min M2	Targeting GABA_B_R Targeting GABA_A_R	[313]

Abbreviations: ↑ enhance or improve with significant differences; ↓ downregulate or inhibit with significant differences; ‐, no significant effects; FSM, Fat/sucrose mixtures; I.P, intraperitoneal; LDX, lisdexamfetaminedimesylate; OA, orally administered; M2, meal 2 for palatable food consumption; SA, self‐administration; S.C, subcutaneous; SD, Sprague–Dawley; WAT, white adipose tissue; WD, palatable western diet.

#### 4.2.1 Effects of GABA_B_Rs Alone on BE

Overeating or BE, an abnormal increase in feeding behavior that is driven by powerful feeding motivations, food craving, or hedonic seeking and exacerbated by negative emotion or excessive stress [13, 36, 52, 64]. BE of sugar, fat, and palatable diets has behavioral and neurochemical similarities to SUDs and may be considered natural addiction or food addiction [13, 26, 314, 315, 316]. In a preclinical setting, activation of GABA_B_Rs reduced drug addiction‐like responses in animal models and showed great promise for the treatment of SUDs in the clinic [100, 317]. Furthermore, activation of GABA_B_Rs generally reduced binge frequency and binge size in animals and humans, decreased fat intake in BE models and inhibited the binge feeding of fat or a palatable diet under non‐food‐deprived and other conditions [308, 309], which was similar to the effects of baclofen on drug SA and SUDs reported by others [45, 318]. Thus, GABA_B_Rs may be important therapeutic targets for BE and food addiction.

When animals were exposed to 12‐h daily access to different chows (sucrose, fat, vegetable–fat, or sweet–fat mixtures) for animal, baclofen (I.P.) suppressed the BE of pure fat during 2 h of access, but increased sweet–fat chow intake and had no effect on sucrose intake [307]. Under binge‐type conditions, the fat‐matched group was provided with chow mixed with fat in the appropriate ratio, and it was found that baclofen (1.0 and 1.8 mg/kg) notably decreased the shortening intake within a 2‐h period; conversely, baclofen significantly increased the chow intake for 2 h [309]. During access to food pellets or vegetable shortening via SA, administration of baclofen (1.0 mg/kg, I.P.) significantly reduced shortening intake relative to saline intake but had no significant effect on pellet intake, and a dosage of 1.8 mg/kg baclofen significantly decreased both pellet and shortening intake for all schedules [308]. Notably, a lower dosage of baclofen was sufficient to significantly reduce shortening intake compared with the dosage needed to reduce food pellet intake.

In a study on binge‐eating like models, baclofen demonstrated a notable reduction in the consumption of semisolid shortening emulsions, but had no effect on the consumption of normal chow [68, 305]. When rats had brief limited access to fat and continuous access to chow, administration of baclofen inhibited the consumption of semisolid vegetable fat (shortening) [303, 304, 307]; in a study in which daily or intermittent access to fat or sucrose mixtures containing 3.2% (L), 10% (M), or 32% (H) powdered sugar in 100% vegetable shortening, administration of baclofen decreased the consumption of the L and M mixtures by male SD rats [304]. In addition, administration of baclofen reduced lard intake under binge‐type and nonbinge‐type conditions when lard was presented alone; decreased shortening intake only under nonbinge‐type conditions and lard intake only under binge‐type conditions [33]. Therefore, the ability of baclofen to reduce fat intake appears to be heavily influenced by the type of food consumed (fat), the presentation mode (one fat alone or two fats simultaneously), and the duration between baclofen administration and consumption.

These results indicate (Figure 4 and Table 3) that pharmacological interventions modulating GABA_B_Rs can reduce some special conditions of BE, such as binge frequency, binge size, and shortening intake under binge‐condition establishments, but show no effects on normal intake (chow). The inhibitory effects of baclofen on food intake appear to be specific to fat, as baclofen has no effect on sucrose intake and the sucrose concentration affects the inhibitory effects of baclofen. And there are also some differences among underlying intervention effects of GABA_B_Rs; feeding bouts of fatty and sugar‐rich foods may prove to be particularly regulated via crosstalk interactions of modulating GABA_B_Rs, opioids and dopamine.

#### 4.2.2 Effects of GABA_B_Rs and Opioid Receptors

As shown in Figure 4 and Table 3, feeding regulation is related to the central reward system, GABAergic neurons, and related receptors, and excess consumption of palatable food significantly affects synaptic functions, plasticity and receptor molecules in reward‐related regions; all of these effects resemble SUD in terms of abnormal seeking‐substance behaviors and neuropathology. Thus, pharmaceutical interventions for SUD might also prove efficacious in addressing overeating, BE and food addiction. It has also been confirmed that baclofen and naltrexone, an opioid antagonist, can treat addiction and suppress the intake of certain foods [26, 302]. When used to selectively target GABA_B_Rs and opioid receptors, these medications have demonstrated greater efficacy in reducing abnormal substance‐seeking behaviors compared with using each medication individually [26, 302].

An overeating/BE‐like behavior model was induced in male SD rats via 12‐h binge access to standard chow, sugar solution, and fat emulsion or sugar–fat mix emulsion for 21 days [26, 302]; administration of both baclofen and naltrexone (I.P.) deceased the intake of palatable food in both the fat and sugar–fat groups, while having no impact on the consumption of normal chow. Furthermore, administration of baclofen alone significantly reduced the consumption of palatable food in the fat and fat–sugar groups, but naltrexone whereas naltrexone had minimal impact on the intake of either palatable food or chow in these groups [26, 302]. In addition, naltrexone decreased the consumption of sugar at higher amounts compared with the amount needed to decrease the consumption of fat, but it did not have a notable impact on the intake of simultaneously available chow [303, 304]. Conversely, naltrexone significantly reduced the intake of 32% and 56% fat emulsions intake on the intermittent schedule but it did not affect the intake of chow [304]; blocking opioid reduced intake of shortening and did not decrease sucrose intake in two of the daily access groups [303, 304]. Consequently, Interventions of targeting GABA_B_R exert vital roles in the regulation of the fat and sugar–fat‐related food intake, irrespective of the access schedule; but it shows little impact on sucrose intake, which is different from the effects of naltrexone in the reducing sucrose intake.

In a BE model, administration of nalmefene exhibited a dose‐dependent reduction in chocolate intake, while it had no effect on 2‐h intake of chow [305]. Similarly, baclofen significantly decreased the chocolate intake with a dose‐dependent manner, but did not affect chow intake [305]. The additional findings indicate that the fat intake‐reducing effects of GABA_B_R‐targeting agents depend on the fat concentration and access schedule to fat [263]. Moreover, SB‐334867 (an orexin‐1 [OX1] receptor antagonist) significantly reduced chocolate intake without significant effects on standard chow intake [305].

Thus, OX1, baclofen and nalmefene have similar effects in inhibiting chocolate intake but not standard chow intake. First, administration of baclofen (GABA_B_R agonist) reduces the intake of fat‐enriched palatable dietary, and naltrexone (opioid antagonist) suppressed the intake of some food. Then, a combination of baclofen and naltrexone is a vital pharmacological strategy for BE and food addiction, as both of these medications are used for alcohol dependence [319] and other addictions [26]. Selectively targeting GABA_B_ and opioid receptors has significant effects in suppressing overeating of palatable foods, high‐fat diets and food craving [64, 320]. From a neurobiological perspective, activation of GABA_B_Rs seems to regulate only high‐fat diet or high‐calorie intake, and the underlying mechanisms are still unclear (Figure 4 and Table 3).

#### 4.2.3 Effects of GABA_B_Rs and D1/2 Receptors

In addition to GABA, opioids and their receptors are implicated in food addiction and BE, and sucrose may alter the effects of dopamine and dopamine receptors (D1R and D2R) on the consumption of fatty food [303, 304], indicating that GABA_B_Rs and D1/2 receptors may be involved in the regulation of BE and food addiction.

In a study, rats were given solid fat emulsion at different concentrations (18, 32, and 56%) either every day or intermittently [304]. The intake of emulsion was significantly decreased by both baclofen and raclopride (a D2 antagonist) in all daily access groups, as well as in the intermittent access group for the 56% fat emulsion. And administration raclopride reduced the intake of the group with daily access to the L mixture, while increasing the intake of the group with intermittent access to the L mixture; it also reduced the intake of both groups given the M mixture, but had no effect on either group given the H mixture. Under limited‐access conditions, raclopride stimulated fat intake in rats in the intermittent access group but had no effect on rats in the daily access group. Nevertheless, raclopride reduced sucrose intake in all groups and had no effect on or partly reduced chow intake [303, 304].

Additionally, GABA_B_Rs [64, 320] and D2Rs [303, 304] are dysfunctional in animals with a history of BE fat and sucrose. Activation of GABA_B_Rs reduced fat intake, had no effect on sucrose intake, and either stimulated or had no significant effect on chow intake, and these effects were further verified in a GABA_B_R knockout animal model [64]. In contrast, DA receptor (both D1R and D2R) blockade consistently decreased consumption of sucrose, while the impact on fat intake varied based on the particular subtypes of receptors being blocked and the access schedule [303, 304].

The above findings validate that a potential therapeutic approach for disorders related to BE and food addiction, could involve targeting GABA_B_Rs, D1R, and D2R simultaneously (Figure 4 and Table 3). In addition, these treatments for hedonic overeating not only provides powerful evidence for patients with BE and food addiction but also implies a shared mechanism that underlies addictive behaviors, a topic that has been extensively debated and acknowledge. Thus, BE affects reward‐related molecular targets, and pharmaceutical treatments for SUDs can also be effective in treating food addiction or overeating‐related disorders.

Overall, targeting GABA_B_R can inhibit the binge feeding of fat or a palatable diet under non‐food‐deprived and other conditions, and attenuate this effect of natural reinforcers or craving for food [307, 308, 309]. Obviously, GABA_B_Rs play a vital role in the regulation of both normal feeding and overeating or BE‐like behaviors. On one hand, the complex effects of GABA_B_R are associated with its multiple interaction with other neuropeptides, neurotransmitters, or related receptors through different mechanisms; on the other hand, the underlying mechanisms are involved in the pathological conditions through different motivational mechanisms, different brain subregional networks, and reactions of different intensities, which needs further in‐depth exploration.

Furthermore, recent studies have shown that leptin increases energy expenditure, and regulates body weight, and its receptors interacts with GABAergic neurons and GABA_B_Rs, alleviating hyperglycemia and regulate energy imbalance [321]. GABAergic neurons and GABA_B_Rs are crucial in the regulation of hypothalamic hormones and receptors (such as leptin and CCK) to achieve energy homeostasis, which is likely to account for the effect of GABA_B_R activation in reducing BE of fat. Hence, GABA_B_Rs may be one of the best underlying therapeutic targets for BE and food addictions (Figure 4). Next, we further discussed the and treatment of targeting GABA_B_R on obesity.

### 4.3 Food Addiction and Obesity

Promising outcomes have been obtained from clinical studies involving baclofen for the treatment of alcohol [322], cocaine [323], and opiate use disorders [102, 103, 105]. For instance, an open‐label study was with an increased dose of 30 mg/day for the remaining 27 days of treatment, and it showed that baclofen reduced alcohol craving and alcohol drinking [324, 325]; a randomized controlled trial investigation found that baclofen reduced alcohol intake and craving in the alcohol‐dependent subjects [326]. Targeting GABA_B_Rs has also been employed for BE behaviors [41, 311]. In addition to being a therapeutic target for SUDs, GABA_B_R may be a promising target for treating food addiction and related syndromes, such as BE and obesity.

In both diabetic and diet‐induced obese mice, the peripheral administration of baclofen produced a significant decrease in food intake and body weight; however, it did not have any notable impact on energy balance in lean control mice [284]. Baclofen treatment significantly decreased NYP expression in the ARC but significantly increased POMC mRNA levels; furthermore, baclofen significantly decreased blood glucose and HbA1c levels, significantly increased plasma leptin levels, and significantly decreased the weight of epididymal WAT [284]. These data imply that GABA_B_R‐targeted regulation may decreases excessive adipose stores via the ARC in only obese subjects when used as a strategy for the treatment of obesity.

In a pilot study of obese patients (seven women and three men, BMI between 31.3 and 41.0 kg/m), baclofen (15 mg/day to 30 mg/day, administered for 12 weeks) exhibited a noteworthy reduction in body weight and serum leptin levels, and suppressed appetite and the desire for sweets, contributing to improvement in hypertension, diabetes, and dyslipidemia, and it did significantly alter the levels of adiponectin, FBS, HbA1c, or serum insulin, HOMA‐IR, serum lipid levels or blood pressure of the patients [310]. Furthermore, in an open‐label trial, women with BED and BN took baclofen (60 mg/day) for 10 weeks, and baclofen notably decreased their BE frequency and was well tolerated; four of the five patients exhibited a great reduction in BE scores on the Food Craving Inventory‐II; compared with placebo, baclofen significantly reduced the binge frequency in BE patients [311, 312].

Given that baclofen reduced appetite and leptin levels, its effects on obesity and food addiction might also be enhanced by diet therapy and treatments that decrease adipose stores [284, 310, 311]. In addition, baclofen may be shown to be more effective in reducing body weight in obese humans or in animal [310], as its related side effects of sedation that impact energy expenditure and partly veils the regulator inhibition for food appetite and weight. Hence, the further detailed assessments are needed via an approach of specially targeting‐GABA_B_R measure.

Preclinical and clinical related studies have indicated that the activity of GABA_B_Rs in the CNS plays a role in the regulation of food intake, rewards, and related peripheral activities, at least partially. Targeting GABA_B_Rs can be developed as a novel therapeutic approach for food addiction and obesity, and the underlying mechanisms of regulation of GABA_B_Rs appears to be associated with inhibition of food craving, reward abnormalities and control‐decision imbalance in feeding behaviors (Figure 4).

### 4.4 Peripheral Regulatory Effects

Additionally, GABAergic system is also present in peripheral tissues [327, 328, 329, 330]. As shown in Figures 1 and 4, GABA_B_Rs are widely distributed along central vagal pathways in the nucleus tractus solitarius (NTS) and dorsal vagal nucleus [280, 328]. Studies have shown that baclofen has effects on physiological vagal inputs to the NTS from other viscera and on electrically stimulated inputs by abdominal vagal afferents [328, 331]. GABA_B_Rs markedly inhibit vagal afferent pathways centrally and peripherally; GABA modulates gastric acid secretion [328, 329, 330] and exerts inhibitory and stimulatory effects on GABAergic system activity in the gastric system in rodents [82, 327, 328] and humans [327, 332, 333].

Administration of baclofen (intravenous, 7–14 mmol/kg) reduced decreased the reactions of gastric tension and NTS neuron responses to gastric distension, but did not affect the responses of gastroduodenal mucosal receptors to CCK; these responses were reversed by the antagonist CGP‐35348, suggesting that the GABA_B_Rs might inhibit the mechanosensitivity, but not chemosensitivity, of peripheral vagal afferents [327, 328].

GABA_B_Rs reduced the central mechanosensory inputs to brain stem neurons while having no impact on the transmission of excitatory chemosensory signals. And they decreased the inhibitory mechanosensory and chemosensory inputs to brain stem neurons originating from inhibitory interneurons, but the pathway of chemosensory inputs involved GABA_B_Rs [327, 328]. Peripheral vagal nerve stimulation via microchip implantation increased vagal afferent activity and thus induced alterations in feeding behaviors, resulting in to a decrease of nocturnal food intake and body weight [280]. However, these effects were partially attenuated by baclofen; baclofen significantly stimulated gastric motility and elicited the formation of an irregular motor migration complex [280], indicating that GABA_B_Rs depend on permanent vagal nerve stimulation. These results suggest that GABA_B_Rs regulate gastric‐related motility and the release and expression of feeding‐related hormones through permanent vagal nerve stimulation (Figure 4).

In addition, baclofen notably affected and regulated feeding behavior and weight [280, 328] via peripheral regulation of GABA_B_Rs and vagal neuromodulation, small intestinal transit suppression, increased gastric acid and gastrin secretion through vagal‐dependent manners and suppressed food intake and small intestinal transit via u‐opioid receptors coupled to the 5‐HT_1A_, D2, and GABA_B_ systems [280, 327, 328].

The peripheral regulation of GABA_B_Rs may affect not only the gastric secretion of CCK, GLP‐1, and ghrelin, but also gastric‐related motility and activity via vagal nerve stimulation, which may result in further interactions with central nervous circuits and networks associated with feeding or eating disorders (Figure 4). As the existence of the brain–gut axis and has been confirmed to have play vital roles in feeding or eating disorders, it is essential to further explore the detailed mechanisms of GABA_B_Rs involved in the processes of brain to peripheral organs. For instance, GABA receptors can control intestinal fat absorption via suppression of the midbrain–vagus–jejunum axis [334].

## 5 Effects in Oncology

In addition to the roles in the various neuropsychiatric disorders, BE, and feeding‐related disorders, GABA_B_Rs are involved in the modulations of the tumour biochemical and pathological processes [335, 336, 337, 338, 339, 340], such as prostate cancer [336], breast cancer [338], and pancreatic cancer [340]. The GABA_B_Rs and GABAergic signaling pathways can inhibit cell proliferation [341, 342, 343], regulate tumor microenvironment [337], and exert vital roles in the tumor metastasis and progression [338, 341, 344]. For instance, baclofen significantly increases cell invasion and migration and mediates breast cancer metastasis via ERK1/2 pathway [338]. In contrast, GABA_B_R antagonists (CGP55845 and CGP35348) decrease tumor cell growth, invasion, and migration, induce cancer cells apoptosis [342, 343, 344, 345], and show antitumor effects via caspase 3/9 apoptotic pathways and PI3/Akt/MAPK pathways [341, 345]. In summary, GABA_B_Rs and their crosstalk with the related signaling pathways play a complex but crucial regulatory role in tumors. However, current neuro‐oncology research focuses on multipathway crosstalk, including tumor‐immune‐metabolic interplay, epigenetic‐genetic synergy, and multimodal therapeutic integration, addressing glioblastoma heterogeneity and resistance mechanisms; those other involvement in complex interplay of neurometabolic and immunity can also affect tumour cell proliferation, apoptosis, angiogenesis, and metastasis, has significant implications for cancer biology, but the literature on these aspects of GABA_B_Rs is relatively limited and is thus not further explored in this review.

## 6 Therapeutical Prospects of Specially Targeting Sites

### 6.1 Overall Therapeutic Effects

This review provides an overview of the preclinical and clinical research on the effects of GABA_B_Rs and analogues on measures of various mental disorders (Table 1) and feeding‐related disorders (including food addiction, BE, and obesity), as well as the potential mechanisms by which GABA_B_R may be involved in brain sub‐regions involved in the complex crosstalk among reward, feeding, motivation, and stress. However, most of this research has focused on common mental disorders and normal feeding behaviors under a normal diet or conditions, and few studies reported are related to special psychiatric disorders, BE, food addiction, and BE‐related obesity. More studies, specifically longitudinal studies involving various stress‐inducing tasks and specific eating‐ and mood‐related questionnaires, are needed to fully assess the therapeutic potential of GABA_B_Rs in stress‐induced neuropsychiatric symptoms, abnormal feeding eating behaviors, and other complicated disorders in humans, and even potentially drug addiction (Figures 3 and 4).

**FIGURE 5:**
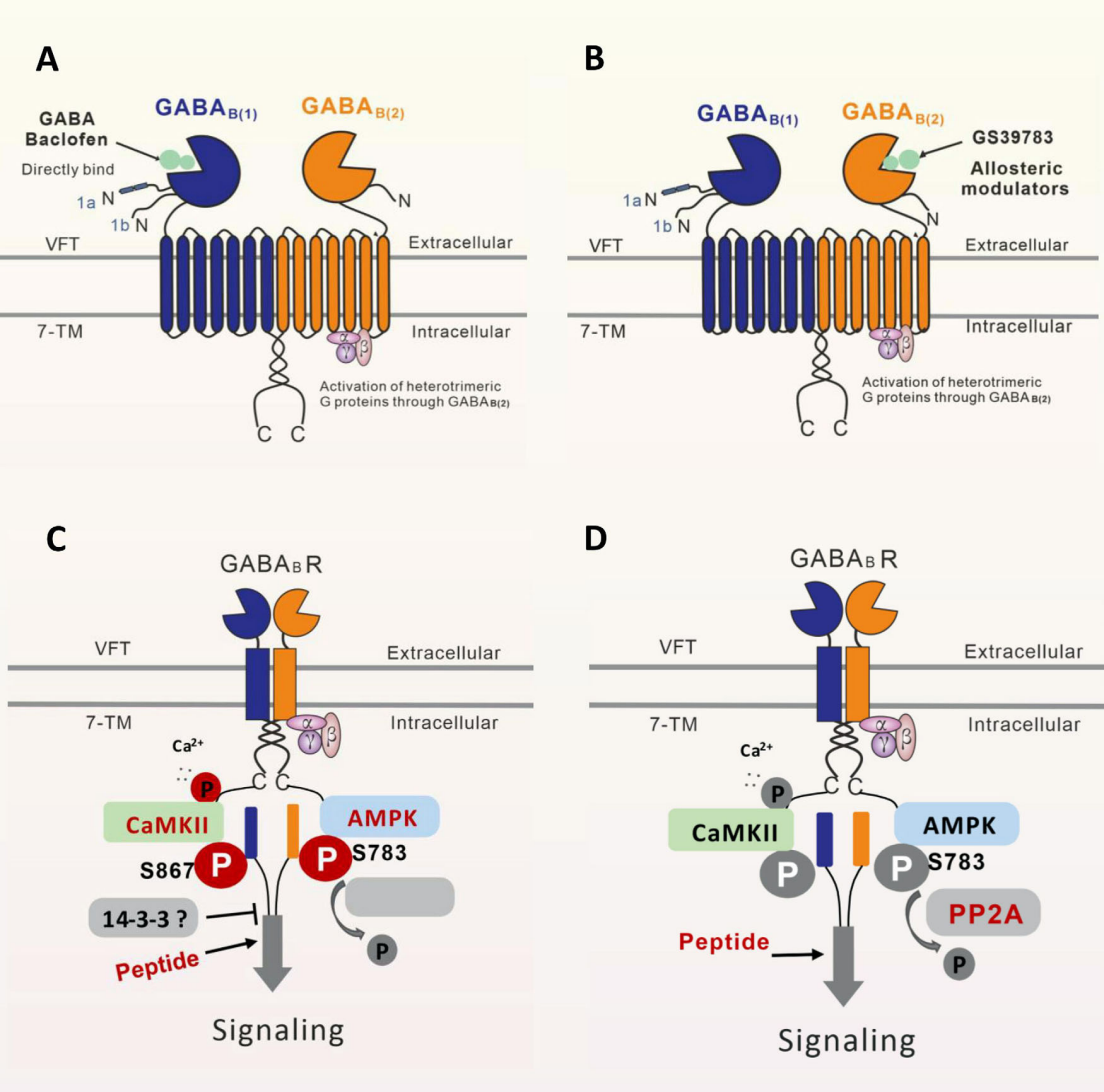
Regulated sites and signaling pathways and therapeutic prospects of strategies targeting GABA_B_Rs for the treatment of binge eating, food addiction and obesity. (A) The orthosteric ligand recognition site of GB1 binds to its ligands such as GABA and baclofen. (B) The allosteric ligand recognition site of GB2 binds to its allosteric modulators, such as GS39783. (C) Potential phosphorylation sites of the downstream GABA_B_Rs, including Ser867 of GB1, which is phosphorylated by CAMKII and Ser783 of GB2, which is phosphorylated by AMPK, and sites in other regulatory 14‐3‐3‐ζ proteins. (D) PP2A regulates downstream GABA_B_Rs via dephosphorylation. Small peptides and compounds exert marked regulatory effects.

Recent studies provide powerful support for GABA_B_Rs as therapeutic targets for neuropsychiatric disorders, BE, food addiction, and obesity, which may involve abnormalities in reward‐related neural circuitry, energy encoding, and reward‐driven decision‐making. However, the side effects of strategies directly targeting GB1 cannot be ignored, as systemically administered baclofen and analogue agonists/compounds can drastically activate GABA_B_Rs and have the same adverse effects as other treatments [86, 305]. Thus, candidate ligands or agents should specifically target GB1 or GB2 or target signaling pathways downstream of GB1/2 and slightly activate them, and be well tolerated (Figure 5A,B).

### 6.2 Orthosteric Ligand Recognition by GB1

The clinical results demonstrate that baclofen has therapeutic promising for SUDs, especially in patients with severe AUD [88, 317]. Despite clinical and preclinical studies have shown positive outcomes for addiction or carving‐like behaviors, the utilization of baclofen is still a subject of controversy and is restricted due to its inadequate clinical safety and adverse effects; baclofen and analogous agents are still not approved by the FDA or EMA for AUD, possibly due to the potential side effects of the compounds.

Baclofen is primarily used as a muscle relaxant in the clinic at a usual dose range of 20–80 mg/day, with few significant side effects [309]. In open human trials, baclofen is well tolerated [310, 346, 347, 348]. Sedation was the commonly reported adverse reaction to baclofen. In the clinic, two patients experienced minor adverse events during a trial; a single individual encountered slight swelling in her lower limbs, likely associated with earlier sunburn [311]. Furthermore, in clinical trials on the use of cocaine and baclofen for treating alcohol‐abusing patients [89, 349], administration of baclofen resulted in the occurrence of symptoms such as headache, nausea, sedation, and dizziness [310, 347, 348, 350, 351, 352, 353].

In addition, baclofen has been reported to cause sedation, muscle relaxation, ataxia in rats, mice, and pigs, and nausea in monkeys [95, 352, 354, 355, 356]. These potential side effects could potentially interfere with the drug's impact on food intake [87]. Baclofen successfully reduced alcohol consumption in mice at a dose that suppressed the reinforcing effects of SA but also suppressed locomotion [89, 349]. Some of the rats presented signs of ataxia approximately 10–30 min after injection (4 mg/kg baclofen) on the first day [279], and it was thought that the ataxia observed in some of the animals might have been interfered with their feeding ability. Furthermore, some sedation was observed in the rats, and these side effects were mild in severity, except at the highest dose tested (10 mg/kg) [305]. Recent studies in animals showed that the 0–4 mg/kg dose of baclofen was safe and well tolerated [63, 64, 85]. The overall discussions and results imply that negative side effects are likely to be cause by a high dose of baclofen, different agonists, or overactivation of GABA_B_Rs.

As GABA_B_Rs are most densely expressed in the cerebral cortex, thalamic nuclei, midbrain, hypothalamus, and cerebellum (Figure 1), but also highly expressed in several other limbic structures, in the spinal cord [89] and even in the gastrointestinal tract. Orthosteric ligands modulate the activity of abundant receptors involved in a variety of brain functions. When the extracellular domain and orthosteric ligand recognition sites of GB1 are directly bound by specific agents or ligands, an active and closed conformation is stabilized by an agonist bound to the VFT domain (Figures 5A and 1), resulting in significant and widespread physiological and pathological regulatory effects through a G‐protein coupling mechanism and signal transduction pathways involving downstream effectors, which is the basis of the negative side effects of GABA and baclofen [74, 75]. It is therefore rather unlikely that the unwanted effects of systemically administered drugs that bind to orthosteric or allosteric binding sites can be avoided.

The selective and potent agonist of the GABA_B_Rs include CGP44532, CGP35024, and baclofen [70, 89]. Baclofen, as a representative ligand that reacts with the key structural domain of GB1 (Figure 5A), has stronger binding affinity, thus excessively blocking neurotransmitter release and inhibiting neuroexcitability [72, 75] in both presynapses and postsynapses through G protein coupling voltage‐gated Ca^2+^ channels, or inducing the hyperpolarization of neurons by opening GIRK channels [71, 74], which may be involved in its underlying side effects, at least partly.

In general, the potential negative side effects may mainly stem from the wide distribution and vital regulatory effects or overactivation of GABA_B_Rs. To avoid these side effects, promising ligands or agents that selectively bind the GABA_B_R subunit involved in a given pathology, do not affect receptors in healthy neurons, slightly activate GABA_B_Rs, and are well tolerated by patients are needed. In the next sections, we mainly discuss the therapeutic effects and the potential side effects that may be induced by the activations of different‐targets or sites.

### 6.3 Allosteric Ligand Recognition by GB2

GABA_B_R activation is proposed to involve a unique allosteric mechanism (Figures 1, 2, and 5B), where binding of an agonist to the VFT domain of GB1 results in a series of conformational rearrangements, which are transmitted to the TMD of GB2 to trigger G protein signaling [71, 75]. In addition to the binding of ligands to GB1, the activity of GABA_B_Rs can be regulated by allosteric modifiers called PAMs, which have analogous efficacy to orthosteric ligands, or negative allosteric modulators (NAMs) [72, 74] via allosteric ligand recognition [70, 74]. Some comparative preclinical studies have demonstrated that NAMs targeting allosteric ligand recognition produce fewer side effects, are better tolerated and are more effective than expected among baclofen and other drugs [70, 85, 88, 99].

For instance, Infusion of the SKF97541 (GABA_B_R agonists) into the PFC and STR significantly reduced basal DA levels, and this effect was reversed by the antagonist CGP52432. Similar to agonists [357], PAMs targeting GABA_B_Rs, such as GS39783 [88, 358], CGP7930 [358], and BHF177 [70, 359], have been repeatedly reported to suppress multiple substance addiction‐related behaviors in tests involving an oral SA apparatus and preference tests [89, 100, 359, 360], including AUD [88, 318, 361], sucrose‐induced food addiction [45, 89], and cocaine seeking [89, 100]. Furthermore, ADX71441 dose‐dependently decreased alcohol SA without causing sedation and prevented reinstatement of seeking and relapse‐like behavior according to mapping of neuronal activation [99]. In addition, it was demonstrated to excellent preclinical efficacy and tolerability in several rodent models [59, 70].

PAMs of GABA_B_R (Figures 2, 3, and 5B), which act by potentiating the signaling resulting from binding to allosteric sites of GB2 rather than binding to intrinsic sites of GB1, offer several advantages over orthosteric ligands, including fewer adverse effects, good tolerance, and better therapeutic efficiency [75, 86, 88, 99]. This suggests that the efficacy of GABA_B_‐targeting PAMs have better therapeutic efficacy for addiction, and own high translational potential in humans and merit clinical testing, particularly in patients. Thus, PAMs may have therapeutic benefit for preventing and treating various psychiatric disorders, food addiction, BE, and feeding‐related metabolism disorders (obesity). However, there are relatively few research results of clinical studies targeting the GB2 of GABA_B_Rs. More basic and clinical studies of are needed in the future to further evaluate and investigate the efficacy and safety of PAMs in the treating assessment (Table 1 and Figure 5).

### 6.4 Downstream Phosphorylation Signaling

In the signaling pathways of GPCRs, phosphorylated regulation are vital functional processes. GABA_B_Rs are phosphorylated by PKA, adenosine 5′‐monophosphate‐activated protein kinase (AMPK), and Ca^2+^‐dependent protein kinase II (CaMKII); the former two stabilize the receptor on the cell surface and the latter promotes receptor endocytosis and degradation. AMPK‐induced phosphorylation facilitates the recycling process, while protein phosphatase 2A (PP2A) triggers dephosphorylation, resulting in lysosomal degradation [74, 362]. Importantly, AMPK and PP2A activity is ideally balanced under basal conditions [85, 94]. Phosphorylation of GB2 at Ser783 can be increased by AMPK and determines receptor fate during postendocytic sorting. Additionally, PP2A‐mediated regulation of GABA_B_R–GIRK signaling has thus far been observed in multiple brain regions in response to various stimuli [85, 94, 363].

Pharmacological inhibition of PP2A ameliorates depression‐like symptoms by improving GABA_B_R–GIRK function and neuronal excitability in a depression model [94]. In layer 5/6 pyramidal neurons of the prelimbic cortex, PP2A‐dependent suppression of GABA_B_R–GIRK, which enhanced cocaine‐induced locomotor activity and inhibited behavioral sensitization and represents an early adaptation that is critical for promoting addiction‐related behaviors, was observed [363]. These results demonstrate that the regulatory effects of PP2A by phosphorylating Ser783 of GB2 may be a potential target for regulating aberrant GABA_B_R signaling through GIRK channels and intracellular constitutive endocytosis of GABA_B_Rs (Figure 5C,D).

Furthermore, CaMKII causes crosstalk between GABA_B_R signaling and glutamate signaling, interacts with NMDA receptors and regulates NMDA‐mediated plasticity [172, 364]. Prevention of the dephosphorylation of GB2 at Ser783 has an added benefit [72, 365]; it supports the phosphorylation of this residue by AMPK under ischemic conditions, and it exerts neuroprotection by stabilizing GABA_B_R functions [365]. It is known that that activity of CaMKII and AMPK is mediated by a rise in intracellular Ca^2+^ levels and a decrease in the ATP/AMP ratio, and CaMKII binds to GABA_B_Rs and phosphorylates GB1 at Ser867 [366], indicating that these effects or inactive regulation maintain glutamatergic excitability stability. Mutational inhibition of Ser867 phosphorylation by CaMKII abolishes the downregulation of GABA_B_R expression. Thus, the interaction of CaMKII and PP2A with GABA_B_Rs maintains normal CNS excitability and inhibitory levels by counteracting overexcitation and excitotoxicity under abnormal conditions or stress [94, 362, 363, 365], as shown in Figure 5C,D.

Identification of the sites at which CaMKII and PP2A interact with GABA_B_Rs would permit the development of synthetic small interfering peptides or agents to disrupt these interactions and prevent phosphorylation of GB1 at Ser867 [85, 94, 363, 366] or dephosphorylation of GB2 at Ser783 [85, 94, 362, 363, 365]. Recent findings indicate that a brief peptide corresponding to the PP2A interaction region site on GB1 competes for PP2A binding, enhances phosphorylation of GB2 (Ser783), and impacts signaling through GIRK channels [362]. Certain brain peptides, like MIF‐1 (Pro–Leu–Gly–NH2) and Tyr–MIF‐1 (Tyr–Pro–Leu–Gly–NH2), amplify GABA‐stimulated benzodiazepine binding on the membrane in the mouse cortex [367]. α‐Conotoxins, a subset of compounds composed of 11–20 amino acid residues, inhibit the GABA_B_R‐coupled N‐type calcium (Cav2.2) channels [368], produces both acute and long lasting analgesia [91], accelerates the recovery of function after nerve injury, and alleviates neuropathic pain; alpha9–alpha10 antagonists are also potent GABA_B_R agonists [91, 368], and phosphorylation of downstream mitogen‐activated protein kinase/extracellular signal‐regulated kinase (ERK)1/2 is regulated by these GABA_B_R agonists [85, 369]. Moreover, GABA_B_Rs may regulate numerous cellular processes by interacting with 14‐3‐3 proteins (phosphoserine/phosphothreonine‐recognition proteins; Figure 5B) [370], which are regarded as key agents for the treatment of numerous neurological diseases involved 14‐3‐3 dysfunction [72, 85, 370]. However, there are few direct science date and tests that demonstrate regular effects of the downstream GABA_B_R signaling pathway and its phosphorylation sites on special psychiatric disorders, BE, and feeding‐related obesity.

Based on the above mechanisms, small interfering peptides can optimize the protein–protein interactions between GABA_B_Rs and G protein‐coupled receptors, including the GABA_B_R–GIRK interaction, the NMDA receptor/GABA_B_R–mGluR1 interaction, and the calcium channel pathway, which may allow these peptides to restore normal receptor function selectively at the site of dysfunction under pathological conditions. On the one hand, restoring functional GABA_B_Rs may serve as a new clinical intervention strategy for CNS diseases involving GABA_B_R dysfunction. On the other hand, these phosphorylation sites (CaMKII and PP2A) and the allosteric modulation of GABA_B_R pathways act as promising targets and have been proposed as targets for the development of lead compounds for CNS disorders, such as SUDs [45, 89, 317, 318]. These small interfering synthetic peptides (Figure 5C,D) or agents specifically targeting these potential sites may provide effective therapies that avoid the possible adverse effects of GABA_B_R agonists. Despite, the implications of phosphorylation sites (CaMKII and PP2A) and the allosteric modulations for neuropsychiatric disorders, BE, and feeding‐related metabolism disorders are needed to be explored and confirmed via preclinical and clinical testing.

## 7 Conclusion

In summary, this work demonstrates that GABA_B_Rs are associated with various physiological processes, neurological and psychiatric disorders, feeding behaviors, BE‐related metabolism disorders, and even oncology. Understanding the role of GABA_B_R provides valuable insights into normal brain function and potential therapeutic targets for different psychiatric disorders, BE, and BE‐related metabolism disorders.

The further discussions imply that novel strategies of specifically targeting GB1 or GB2 or downstream signaling pathways of GABA_B_Rs may be effective in treating psychiatric disorders and equally efficacious in attenuating BE and BE‐related metabolism disorders. Within the GABAergic system, GABA_B_Rs are potential therapeutic targets for dealing psychiatric disorders, multiple metabolic, and behavioral disorders. Allosteric modulators and small peptides targeting GABA_B_R signaling pathways, including protein phosphatase 2A (Ser783), CaMKII (Ser867), and 14‐3‐3 proteins, which were shown to have therapeutic effects in preclinical studies, may be developed as attractive drug candidates that avoid the side effects of agents that directly bind these receptors.

However, there is limited preclinical and clinical evidence directly verifying that strategies targeting phosphorylation sites and the allosteric modulation of GABA_B_R pathways (Figure 5C,D) are safe and effective potential methods for treating various psychiatric disorders, BE and obesity (Figures 1, 3, and 4). It remains unclear whether the central and peripheric effects and mechanisms of strategies targeting GABA_B_R pathways are confounded by BE, environmental stress, and abnormal diet‐induced obesity.

Overall, future studies should focus on exploring and confirming the clinical efficacy and safety of PAMs and small interfering peptides used in the treatment of different psychiatric disorders, BE, and BE‐related metabolism disorders. It is necessary and vital to further explore the underlying mechanisms of strategies targeting GABA_B_Rs in reducing BE of palatable diet, which will provide some clues to the therapeutic potential of these strategies for special psychiatric disorders, such as BE, addiction, and BE‐related obesity.

## Author Contributions

W. X. designed the review. W. X. and Y. L. wrote the manuscript. X. W., E. B., E. K., and M. V. helped create the figures and discussed and revised the manuscript. J. H., T. Y., J. C., H. W., and X. C. were mainly responsible for the supervision of the work and the management of related projects. All authors discussed, edited, and approved the final version.

## Ethics Statement

The authors have nothing to report.

## Conflicts of Interest

The authors declare no conflicts of interest.

## Data Availability

The authors have nothing to report.
